# Innovative Tool for Improving Surface Quality in Single Point Incremental Forming: A Comparison with Hemispherical Tools

**DOI:** 10.3390/ma18184275

**Published:** 2025-09-12

**Authors:** Emanuel Bădulescu, Eduard Laurenţiu Niţu, Daniela Monica Iordache, Claudiu Bădulescu

**Affiliations:** 1 Doctoral School of Industrial Engineering and Robotics, National University of Science and Technology Politehnica Bucharest, 060042 Bucharest, Romania; emanuel.badulescu@stud.fiir.upb.ro; 2 Faculty of Mechanics and Technology, National University of Science and Technology Politehnica Bucharest, 060042 Bucharest, Romania; daniela.c.iordache@upb.ro; 3 Dupuy de Lôme Research Institute (IRDL)—UMR CNRS 6027, ENSTA, F-29200 Brest, France; claudiu.badulescu@ensta.fr

**Keywords:** SPIF, AA6061, truncated cone, innovative tool, hemispherical tool, roughness, microhardness, material anisotropy

## Abstract

Single Point Incremental Forming (SPIF) has emerged as a flexible and cost-effective technique for producing complex sheet metal parts. However, its industrial application is often limited by issues related to surface quality. This study examines the impact of tool geometry on the surface integrity of the AA6061-T6 aluminum alloy. The research novelty lies in the innovative eccentric tool with a variable radius (ETVR), which we compare to two conventional hemispherical tools with radii of 5 mm and 10 mm. Truncated cones were formed under the same process conditions, and their quality was assessed by measuring surface roughness and microhardness along the cone’s generatrix in both the rolling direction and the transverse direction. Additionally, microchip analysis and visual inspections were conducted. The results reveal distinct differences in the surface morphology, evolution of roughness, and distribution of microhardness among the three tools. The SS5 tool produced the highest level of hardening but also resulted in significant surface deterioration. In contrast, the SS10 tool generated smoother surfaces with moderate hardening, while the ETVR tool struck a balance between surface uniformity and enhanced hardness. Statistical analyses, using *t*-tests, confirmed the significance of these findings. This study offers new insights into tool design for SPIF, highlighting the trade-offs between surface quality and material strengthening.

## 1. Introduction

### 1.1. Single Point Incremental Forming

Single Point Incremental Forming (SPIF) is an innovative cold forming process, developed over the last two decades as a flexible alternative to conventional deep drawing [1,2,3,4,5,6,7]. The process is based on the progressive deformation of the workpiece under the action of a hemispherical tip tool, which follows a programmed trajectory, typically controlled by CNC machines. This method allows for the production of pieces with complex geometries at low costs, without requiring special molds, making it attractive for prototyping, one-off production, and small series. Due to its high flexibility and low manufacturing costs, SPIF is utilized in a wide range of industrial applications, from rapid prototyping in the automotive and aerospace industries to the production of components in the medical, maritime, and household appliance sectors [1,2,3,4,5,6,7,8,9,10].

### 1.2. Literature Review

Among the materials researched in SPIF, the AA6061-T6 alloy is of particular interest due to its good mechanical properties, low weight, and corrosion resistance, being frequently used for the manufacture of components in the automotive, maritime, and light metal construction industries. However, in the heat-treated state (T6), its deformability is reduced, requiring the appropriate choice of process parameters [11,12,13,14,15].

The conclusions of the study conducted by Sharma et al. [6] highlight the technological difficulties of the SPIF process in the case of heat-treated aluminum alloys, such as AA6061-T6, emphasizing the influence of piece geometry on crack occurrence and the need for solutions to control plastic deformation.

Seyyedi et al. (2023) [12] demonstrated that AA6061-T6 is prone to cracking in SPIF, even in the case of a simple conical geometry, due to local hardening of the material and its low ductility. Several studies have highlighted the sensitivity of AA6061-T6 to process parameters, including tool diameter, incremental step, feed rate, and tool path [10,16,17,18]. It was shown that larger tool radii and optimized forming steps reduce thinning and improve surface finish, while severe local deformation can significantly increase micro-hardness (up to ~175 HV). These results confirm the importance of parameter selection, but also underline the limitations of conventional hemispherical tools.

These results highlight the pronounced sensitivity of the AA6061-T6 alloy to process parameters, demonstrating the importance of deformation conditions in simultaneously improving surface properties and structural integrity in the SPIF process.

Despite its multiple advantages, the industrial use of the SPIF process is still limited by the surface quality obtained and the geometric accuracy of the part. Common problems include high roughness (Ra > 3 µm), excessive wall thinning, and dimensional accuracy. These shortcomings are influenced by process parameters such as vertical pitch, feed rate, rotational speed, tool path, and tool geometry [6,16,17,18].

Although several studies have highlighted the influence of the trajectory on the surface quality and the deformability limit in the SPIF process applied to aluminum alloys, the geometry of the tool used was generally kept constant, having a standard hemispherical shape with diameters between 5 and 16 mm [1,4,16,17,18,19,20,21,22,23], rarely the geometric shape of the tool was varied [15,24].

Studies on various aluminum alloys [20,23,25] have consistently shown that smaller tool diameters (≤6–8 mm) concentrate stresses, leading to cracks and higher roughness, whereas larger diameters (10–16 mm) improve stress distribution, surface quality, and forming depth. Lubrication and tool–sheet interaction mode (sliding vs. rolling) also play a crucial role in controlling roughness.

In the case of low-ductility alloys, such as AA7079, it has been shown that the tool diameter is the main factor influencing the surface roughness, followed by the feed rate and the depth of the step. The use of tools with large diameters (up to 16 mm) led to a significant reduction in the roughness, obtaining a minimum value of 1.91 µm. This behavior is explained by the extension of the contact surface and the reduction of point pressure, which favors a uniform deformation and the decrease in trajectory traces. Therefore, the careful choice of tool size and process parameters is essential to obtain high-quality surfaces in SPIF [26].

In another study [27], the incremental deformation behavior of the aluminum alloys AA1050, AA6061-T6, and AA7075-T6 was analyzed, using ellipsoidal-ended tools with diameters of 6 mm, 8 mm, and 10 mm. The results showed that the tool radius significantly influences the deformability, especially for the AA1050 alloy, where tools with small radii (R = 3 mm) allowed to reach a maximum deformation angle, for AA6061-T6, the highest deformability was obtained with a radius of 4 mm, and for AA7075-T6, tools with large radii led to a significant reduction in the maximum forming angle due to increased forces and the risk of cracking. This variation in behavior is determined by the differences in ductility and stress sensitivity of each alloy, which requires careful adaptation of the tool size depending on the material used.

Dodiya et al. (2020) [15] investigated the effect of tool shape on surface roughness and thickness variation of AA6061 sheet metal, using three tools with the same diameter of 10 mm (one with a flat head, one with an ellipsoidal head, and one with a hemispherical head). The results revealed that the hemispherical head tool provides the best results in terms of external surface roughness, due to more uniform contact and gradual pressure distribution. In contrast, the flat head tool led to lower internal surface roughness, but with possible adverse effects on deformation uniformity. The ellipsoidal tool had the worst performance in terms of internal surface roughness (the highest Ra of all 3 tools, between 3.86 μm and 5.57 μm). Nevertheless, it generated acceptable values for external surface roughness, although lower than those obtained with the hemispherical tool.

Năsulea et al. [24] demonstrated that tool shape adaptation and trajectory compensation can enhance the dimensional accuracy of parts produced through SPIF. The use of a tool with a milled head led to better dimensional accuracy, especially in areas with inclined walls, while trajectory compensation allowed for the correction of deviations in the lower region of the pieces. The results demonstrated that these combined measures can significantly enhance the process without requiring significant modifications to the equipment or process parameters.

The review studies conducted by Trzepiecinski et al. [28], Kumar et al. [29], and Behera et al. [14] highlight that tool geometry influences both forming forces and surface quality. In contrast, eccentric or asymmetrical tools remain poorly documented in the literature. Most works focus on analyzing the influence of tool diameter on material thinning, roughness, and stress distribution, without investigating the effects of unconventional geometries.

The literature review concerning the SPIF process applied to aluminum alloys emphasizes the significant influence of tool geometry on deformation behavior, surface quality, and thickness variation. Most studies have concentrated on tools with hemispherical heads, sometimes compared with flat or ellipsoidal shapes, with variations in diameter. These investigations have demonstrated that both the diameter and the shape of the tool head affect forming forces, surface roughness, and formability, particularly in the case of heat-treated alloys or those with low ductility. However, it is noteworthy that alternative tool shapes, such as those with variable radii or asymmetrical profiles, have not yet been studied.

Among the analyzed works, only the study conducted by Singh et al. [25] explicitly reports the initial roughness values in both the rolling and transverse directions, highlighting a significant difference between them (5%). In contrast, most articles omit entirely any reference to the orientation of measurements with respect to the rolling direction, even though roughness evaluation is used as an indicator of surface quality. This omission represents a gap that leads to incomplete interpretations regarding the effects of the process on the resulting surface quality and microhardness.

### 1.3. Objective and Importance of Our Study

The goal of this study was to assess the surface quality of the parts produced by SPIF using an innovative tool. The innovative tool, named the Eccentric Tool with Variable Radius (ETVR), was designed to improve the Single Point Incremental Forming (SPIF) process. Hemispherical-head tools are regarded as reference tools due to their advantages [30]. Most newly developed tools are compared against this type to emphasize the benefits and drawbacks of the proposed solutions [30].

The parts to be formed by SPIF are truncated cone-shaped components, made of AA6061-T6 aluminum alloy. The AA6061-T6 alloy is notable for its low weight, good mechanical strength, and high corrosion resistance. It is extensively used in various industries, including automotive [31,32], aerospace [33,34,35], railway [31,36], and naval sectors [37,38], making it highly relevant for the cold forming of exterior and structural components [39]. Components made from this material often exhibit intricate three-dimensional concave or convex surfaces with tight tolerance specifications, depending on their functional role within the assembly.

The comparative evaluation of the surface quality includes an analysis of the microchips generated during processing, the visual appearance of the formed part, surface roughness, and the microhardness of the surface layer. Roughness and microhardness were measured in different areas along the cone’s generatrix, in both the rolling direction (RD) and the transverse direction (TD) of the sheet. To compare the quality parameters of the formed parts with those produced by the three different tools, which are linked to specific areas of the cone’s generatrix and various orientations of the sheet (RD and TD), statistical analyses were performed.

The novelty of this study is in the use of the innovative ETVR tool, which combines the advantages of conventional flat-head and hemispherical tools. This design creates variable interaction with the blank surface throughout the process. This approach, which has been rarely explored in the literature, aims to reduce the formation of microchips while simultaneously improving surface quality and uniformity of deformation. The study makes an original contribution by showcasing the benefits of this unconventional geometry, providing new insights for optimizing the SPIF process applied to alloys with limited formability, such as AA6061-T6.

## 2. Materials and Methods

**Figure 1:**
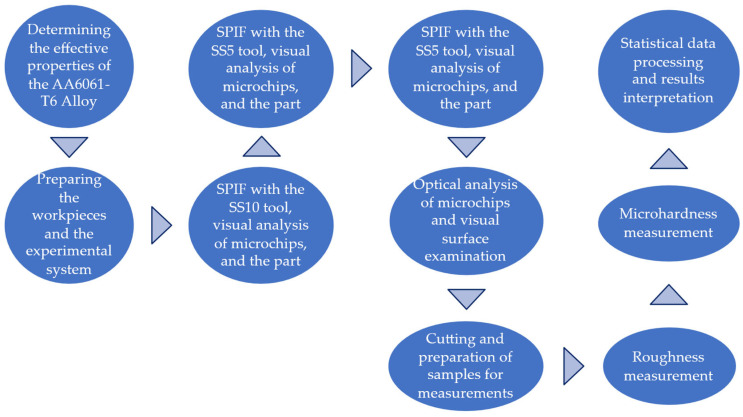
Flowchart of the research activities.

The diagram illustrating the flow of the main activities carried out in this research is shown in Figure 1. The main characteristics of these activities are presented below.

*The chemical composition of the AA6061-T6 alloy* used in this research was analyzed using a Spectro Midex M (Kleve, Germany) energy-dispersive X-ray fluorescence spectrometer. A sample measuring 20 mm × 20 mm × 1 mm was examined, and the findings are summarized in Table 1.

*The mechanical properties of the AA6061-T6 alloy*, as specified in the UNS A96061 standard [40], along with the effective mechanical properties determined by tensile testing of specimens positioned at 45° to the rolling direction of the sheet, are presented in Table 2.

**Figure 2:**
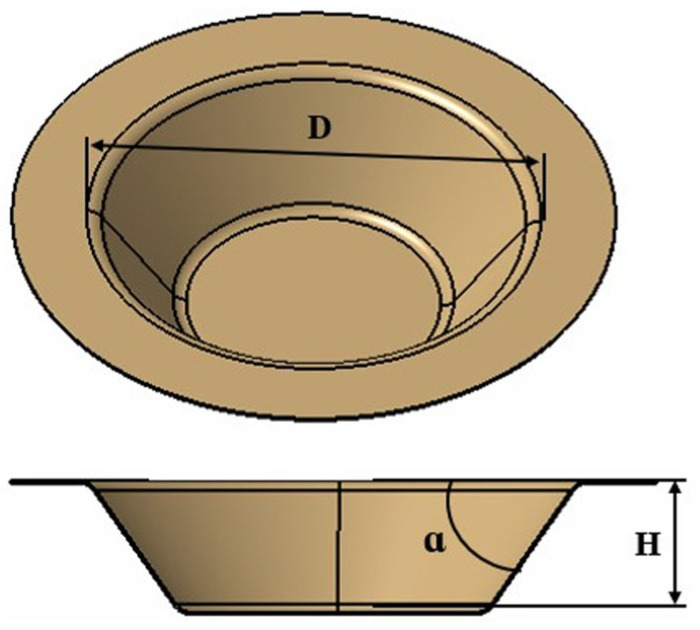
The part to be made through SPIF.

*The objective part* of this research consists of a truncated cone shape created through single-point incremental forming, as illustrated in Figure 2. The main geometric characteristics of the truncated cone are as follows: the large diameter (D) is 96 mm ± 0.5 mm, the height (H) is 25 mm ± 0.5 mm, and the angle of inclination of the wall (α) is 45° ± 1°.

**Figure 3:**
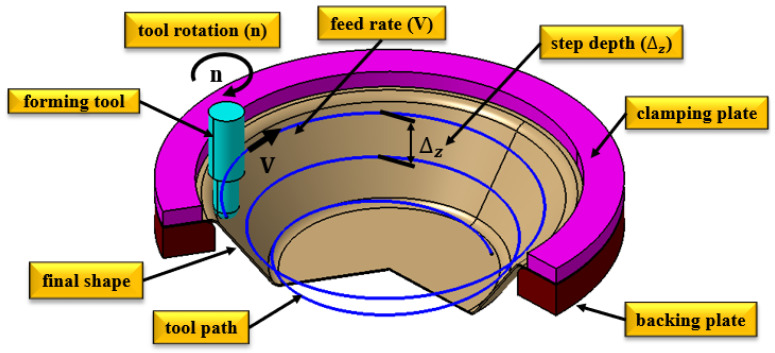
The basic principle of the single point incremental forming (SPIF) process.

The dimensions D, H, and α were established to ensure that the part could be produced using various tools without causing cracks or material breakage. Additionally, this approach would minimize processing time while allowing for the measurement of specific characteristics that define the part’s quality. The single-point incremental plastic forming scheme used in this research is illustrated in Figure 3.

*The technological parameters* for the incremental deformation process are as follows: tool speed (n) in rotations per minute (rot/min), tool feed rate (v) in the XOY working plane (mm/min), and the incremental step of the tool in the Z axis (Δz) (mm). The values for these parameters were determined through an analysis of the literature [18,27,41,42], and were consistently applied in the conducted experiments. Specifically, the tool speed was set at n = 2000 rot/min, the tool feed rate at v = 1000 mm/min, and the incremental step in the Z direction was Δz = 0.3 mm.

**Figure 4:**
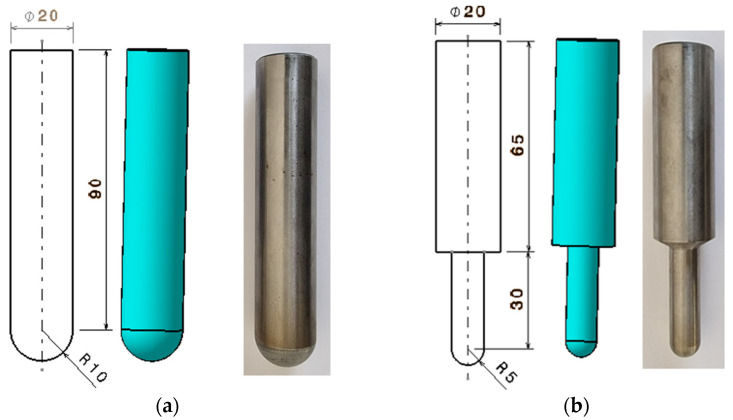
Hemispherical-headed punches used in experimental research: (**a**) punch with hemispherical head with R = 10 mm; (**b**) punch with hemispherical head with R = 5 mm. Unit (mm).

**Figure 5:**
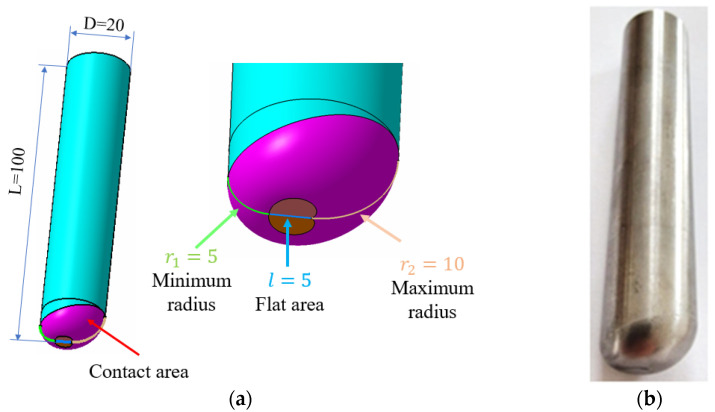
Innovative tool “Eccentric Tool with Variable Radius” (ETVR) [30]: (**a**) 3D model; (**b**) photo. Unit (mm).

To assess the impact of the tool’s shape on the quality of the formed surfaces, *three tools* were employed. All tools were made from the same material—tool steel 1.2312 (40CrMnMoS 8-6)—and shared similar geometric and dimensional characteristics. The tools are as follows:

- A tool with a hemispherical head and an active zone radius of 10 mm, referred to as SS10 (Figure 4a).
- A tool with a hemispherical head and an active zone radius of 5 mm, referred to as SS5 (Figure 4b).
- An innovative tool featuring an eccentric head with a variable radius ranging from 5 to 10 mm, developed by the authors, referred to as ETVR (Figure 5).

*The hemispherical-head tools* with active area radii of 10 mm and 5 mm were selected to examine how the radius affects the quality of the formed surface. The innovative tool, ETVR, features a flat-headed punch with a variable connection radius between the front flat surface and the outer cylindrical surface. This radius ranges from a minimum of 5 mm at one end to a maximum of 10 mm at the opposite end, spanning 180° around the axis, as illustrated in Figure 5. The sizes of these two radii were chosen in relation to those of the hemispherical head tools to facilitate a more precise analysis of the advantages and disadvantages of using all three tools.

*The ETVR tool* combines features of three different tools:

- Flat punch: This tool enhances the material’s deformability by increasing the temperature at the contact area between the tool and the sheet due to intense friction in the flat area.
- Large-radius hemispherical punch (R = 10 mm): This punch positively affects surface quality, resulting in reduced roughness and increased productivity [43].
- Small-radius hemispherical punch (R = 5 mm): This punch also improves material deformability due to its smaller contact area.

A key feature of the innovative ETVR tool is its variable geometry in the active area. As the geometry changes, the size of the contact surface between the tool and the sheet continuously shifts, depending on the angular position of the tool relative to its axis of symmetry and the workpiece. When the rotation speed of the tool (n) exceeds its feed rate (v)—a common scenario in practice—this variation in the active zone creates intermittent forming in the radial direction with each complete 360° rotation of the tool. This can lead to enhanced material deformability [24,44].

**Figure 6:**
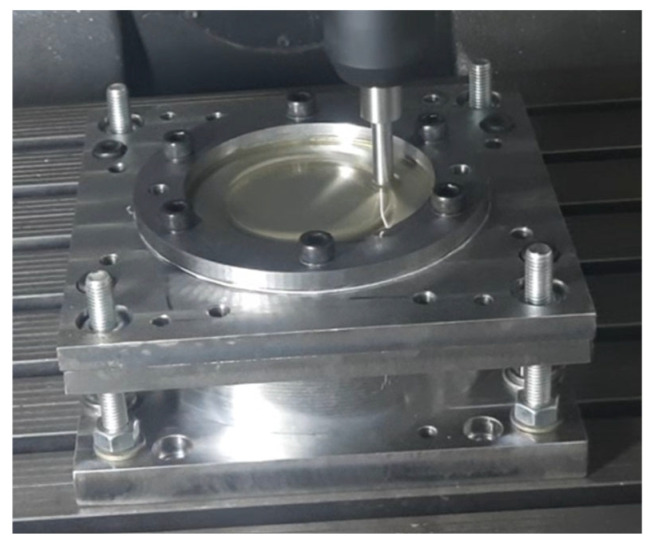
Technological system for processing by SPIF.

*The experiments* were conducted using a numerically controlled milling machine, the EcoMill70 by DMG MORI (Leonberg, Germany) [45]. This machine is equipped with a specialized device for positioning and securing the workpiece. The technological processing system, as shown in Figure 6, features a robust design that resembles systems used in industrial settings.

**Figure 7:**
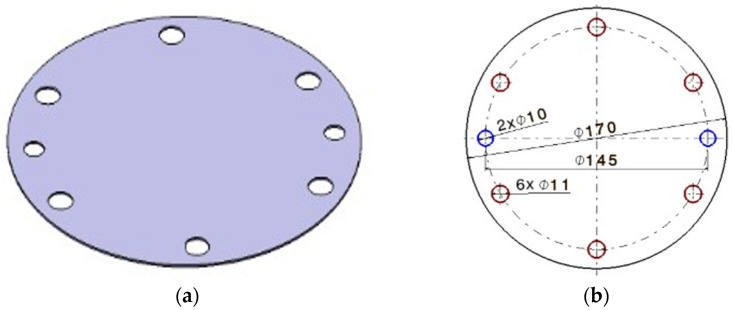
The shape and dimensions of the blank: (**a**) 3D model; (**b**) dimensions of the workpiece. Unit (mm).

*Circular-shaped blanks* with an outer diameter of 170 mm were used, as shown in Figure 7. These blanks were cut from a 1 mm thick laminated sheet using a DURMA HD-F 3015 4 kW (Durmazlar Makina, Bursa, Turkey) laser cutting machine. Each blank has two holes with a diameter of 10 mm, which serve as orientation points for the orientation and fixing device, aligned with the rolling direction of the sheet. Additionally, there are six through-holes with a diameter of 11 mm that allow for the passage of tightening screws for securing the blank.

**Figure 8:**
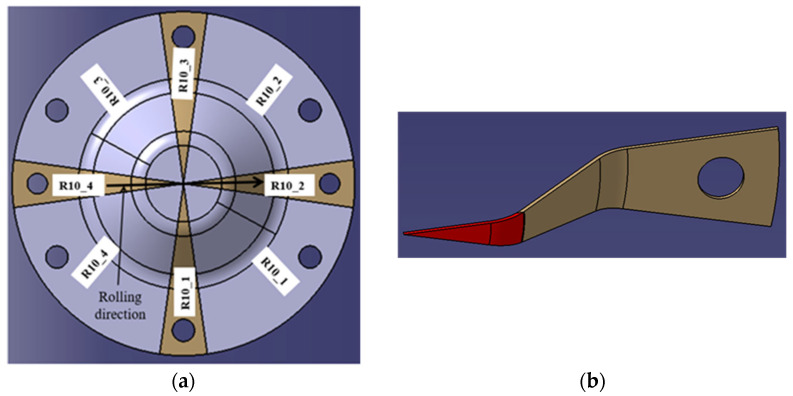
Samples for measuring roughness and microhardness: (**a**) Outline for sample cutting and coding; (**b**) Removing the sample tip to facilitate measuring quality parameters of formed parts.

*The samples used to measure surface roughness and microhardness* were cut using wire electroerosion to prevent any thermo-mechanical alteration of the cutting area, as illustrated in Figure 8a. The cutting planes were positioned to contain the symmetry axis of the piece, ensuring that the small samples used for measurement (see Figure 8b) were symmetrically aligned with respect to the rolling direction (RD) and the transverse direction (TD).

The samples were labeled according to the type and radius of the tool used for processing, with numbers 2 (E2) and 4 (E4) designating the rolling direction and numbers 1 (E1) and 3 (E3) for the transverse direction. To facilitate the microhardness measurement and avoid interference from the rotating head of the microhardness tester, the tip of the sample shown in red in Figure 8b was removed.

**Figure 9:**
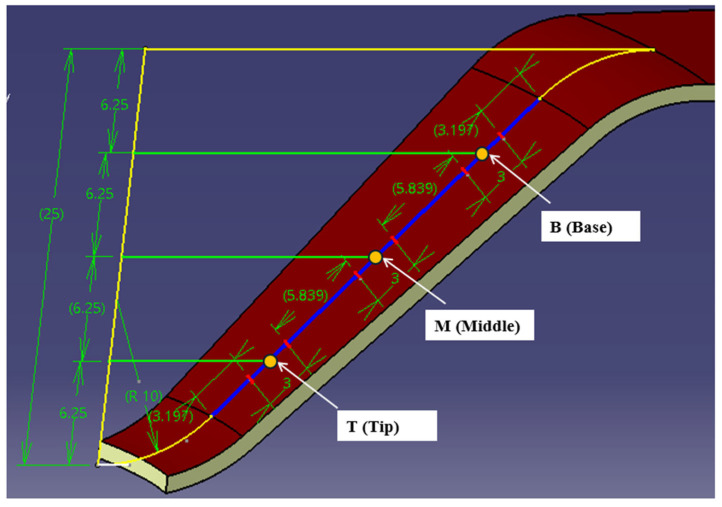
Measurement areas for roughness and microhardness parameter.

For measuring roughness and microhardness, three specific locations on the generator of the formed part were designated: B (Base), M (Middle), and T (Tip), as shown in Figure 9. These locations were established by dividing the height of the truncated cone, which is 25 mm, into four equal segments of 6.25 mm each. On the generator of the part, the areas of interest are marked as follows: at 1/4, 1/2, and 3/4 of the height, a line is drawn perpendicular to the axis of the part, intersecting the generator of the conical surface (indicated by a blue line). This results in the identification of points B, M, and T.

**Figure 10:**
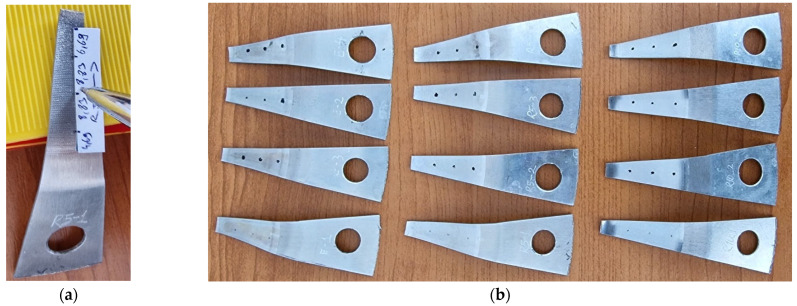
Samples used for measuring roughness and microhardness parameters: (**a**) marking measurement areas using a template; (**b**) images of the 12 samples used for measuring roughness and microhardness.

To ensure accurate positioning of the instruments used to measure the two parameters in the designated measurement areas, a template was created. This template was used to mark the established regions on the sample generator with a marker, as shown in Figure 10a. The twelve samples, obtained by cutting three pieces processed with three different tools, are displayed in Figure 10b.

**Figure 11:**
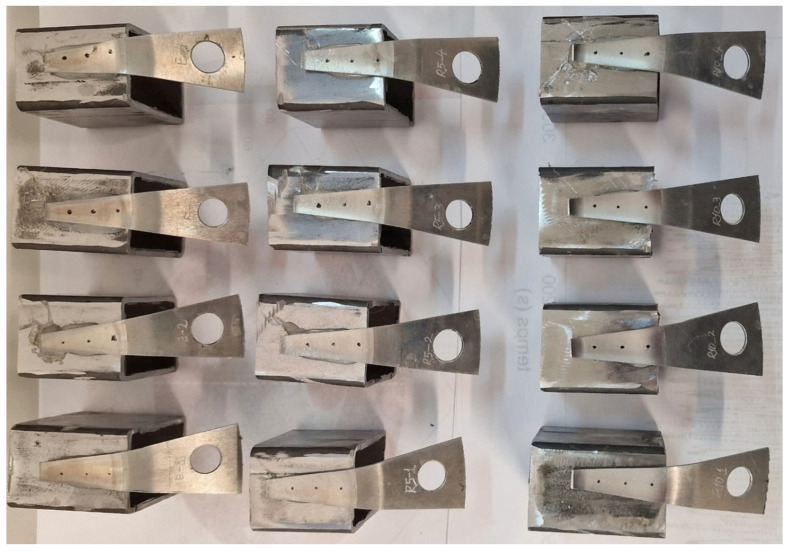
Images of the 12 samples fixed on a rigid support for roughness and microhardness measurement.

After the optical analysis of the surfaces of the 12 samples, they were rigidly attached to rectangular metal supports using a hot-melt adhesive, as shown in Figure 11. The samples were fixed on their sides, ensuring that the convex outer surface made contact with the support. This setup was used for measuring roughness and microhardness.

*The surface roughness* of the blank (rolled sheet) and the parts processed through SPIF was measured according to ISO 4287:1997 [46] and ISO 21920-3:2021 [47] standards. This was carried out using a MITUTOYO SJ210 (Mitutoyo, Kawasaki, Japan) roughness meter [48]. The measurement parameters are detailed in Table 3. Data were recorded directly using a Gaussian filter and automatically compiled into a report in Excel format, thanks to the dedicated SURFTEST program that accompanies the roughness meter.

Among the measured parameters, the analysis focused explicitly on the roughness parameters Ra and Rt. The Ra parameter represents the arithmetic mean of the absolute deviations of a surface’s roughness profile from the mean line along the evaluation length. This parameter was selected because it is the most widely used measure for assessing surface roughness, making it essential in many industrial applications for defining and evaluating the quality of surface finishes. On the other hand, the Rt parameter indicates the maximum distance between the highest peak and the deepest valley on a surface profile over the entire evaluation length. It was chosen because it effectively highlights point defects, such as scratches, material tears, or extreme asperities. This parameter is crucial for parts subjected to high stresses, as significant differences between the maximum and minimum points can lead to the propagation of cracks.

**Figure 12:**
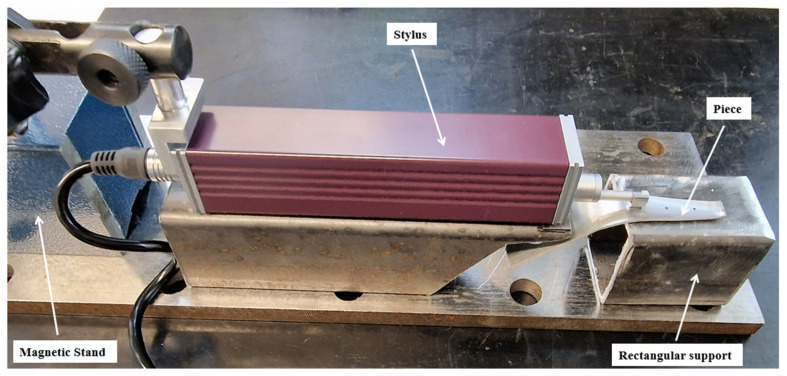
Stand for measuring the roughness of the formed surfaces.

The roughness of the blank (laminated sheet) was measured in two specific directions related to its production: the rolling direction (RD) and the transverse direction (TD), with measurements taken at five points within each direction. To assess the roughness of samples cut from processed parts, the rectangular support holding the sample was placed on a metal base. A mechanical probe was aligned on an additional metal support to ensure it was at the same height as the part’s generator on which the roughness measurement was taken, as shown in Figure 12. The measurements were performed on five segments positioned parallel to the rolling direction, respectively, with the transverse direction, in the case of the workpiece, with the generator of the cone, and centered in the areas of interest marked on the samples (B, M, and T), in the case of the 12 samples.

*The microhardness* of the surface layer of the blank (rolled sheet) and the formed surfaces was measured using the Vickers method, applying a pressing load of 300 gf.

The following considerations were taken into account when selecting the Vickers measurement method and the pressing load:

- The depth of the indentation (h) must be less than 1/10 of the thickness of the sample being studied. Since the smallest wall thickness of the formed part is approximately 0.6 mm (as measured with a micrometer), the indentation depth must not exceed 0.06 mm.
- In the Vickers method, there is a relationship between the depth (h) and the diagonal (d) of the indentation, where h ≈ d/7. Given the maximum indentation depth of 0.06 mm, the maximum diagonal of the indentation should be no more than 0.42 mm.
- Two different pressing loads were tested on the sheettype workpiece: 200 gf and 300 gf. For the 300gf load, diagonal values of approximately 0.06 mm were achieved, meeting the previous requirements. This load was established as preferable because it produces higher imprint diagonal values, facilitating measurement and enhancing the homogeneity of results.
- A pressing load greater than 300 gf was not used, even though it was acceptable concerning the indentation depth. Employing a heavier load would have resulted in a length that would be too large to investigate in the specific areas of the cone generator (B, M, T) while maintaining the condition for the successive positioning of two imprints (l > 6d).

**Figure 13:**
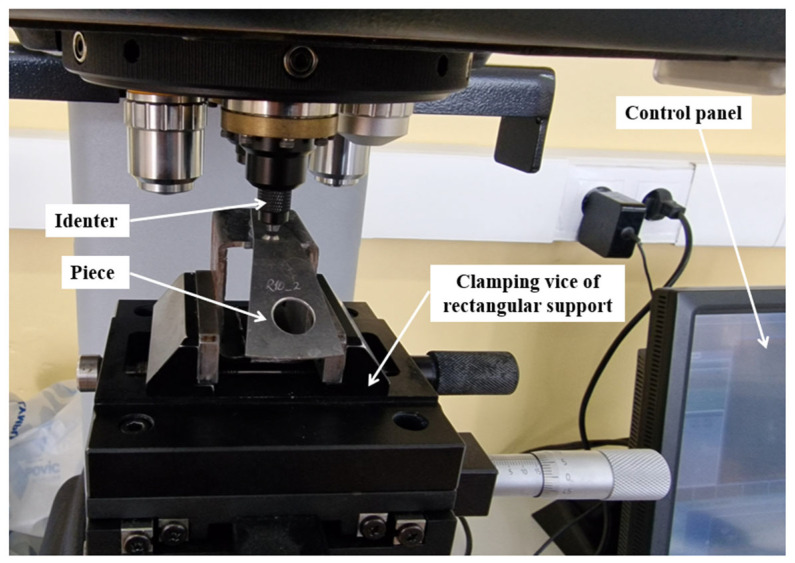
Microhardness measuring stand.

The microhardness (HV) of the blank (laminated sheet) was measured in two specific directions: the rolling direction (RD) and the direction perpendicular to it (TD). Measurements were taken at five points for each direction. Figure 13 illustrates the stand used for measuring microhardness in the superficial layer of the 12 samples. This setup includes a micro-Vickers hardness tester, a rectangular support to secure the sample, and a vice for holding the sample holder in place. The hardness tester, INNOVATEST model Falcon500 (Maastricht, The Netherlands) [49], has a closed-loop force application system with an error of <0.25%, *Z*-axis travel with micron accuracy, and positioning with repeatability within 3 microns.

**Figure 14:**
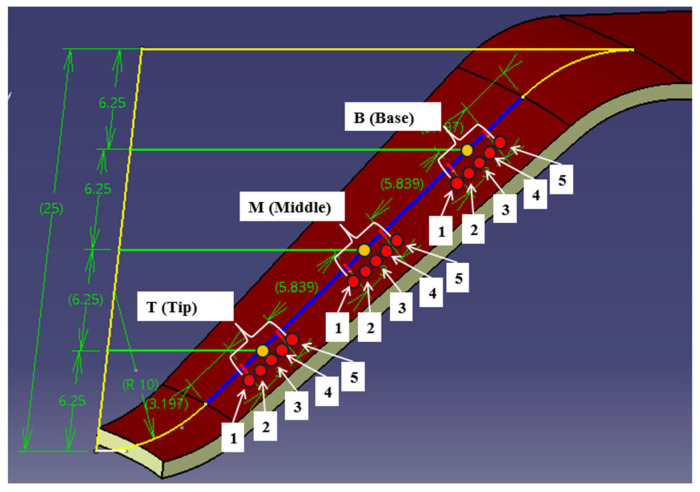
Arrangement of microhardness measurement points in designated areas of samples cut from parts.

The operating parameters during measurement were established according to ASTM E384-11 [50] and included a pressing load of 300 gf, an indenter penetration speed of 0.07 mm/s, and a pressing time of 10 s. In the areas of interest (B, M, and T) of the samples, five microhardness measurements were conducted at points spaced 0.8 mm apart, as shown in Figure 14.

The experimental research plan to measure the quality parameters Ra, Rt, and HV of surfaces formed by SPIF using the three tools is presented in Table 4.

The data were mathematically processed by determining the average values of the surface quality parameters, along with relevant statistical quantities, and analyzed individually. The following quantities were calculated:

- The arithmetic mean of the values (average value), denoted as *X_m_*:

(1) $$X_{m}=\frac{1}{n}\cdot \sum_{i=1}^{n}X_{i}$$

- Standard deviation, denoted as s:

(2) $$s=\sqrt{\frac{\sum_{i=1}^{n}{\left(X_{i}-\;X_{m}\right)}^{2}}{\left(n-1\right)}}$$

- Relative Standard Deviation, denoted as *RSD*:

(3) $$RSD=\;\frac{s}{X_{m}}\cdot 100$$

- Margin of Error, denoted as *ME*:

(4) $$ME=\;t(1-\alpha /2,\;d_{f})\cdot \frac{s}{\sqrt{n}}$$

where

- *X* represents, as the case may be, roughness parameter Ra or Rt, or microhardness HV;
- *n* represents the number of measurements in the analyzed sample;
- *α* represents the significance level associated with the considered confidence level, of 95% (*α* = 0.05);
- $d_{f}$ represents the number of degrees of freedom of the considered data sample.

Statistical analysis was conducted to compare the roughness parameters Ra and Rt, as well as microhardness HV, obtained using different tools. To achieve this, a two-sample *t*-test was employed, which assesses the statistical significance of the differences between the means of two groups of experimental data (the samples being analyzed) [51]. The type of *t*-test chosen for the two independent samples was determined based on the variance ratio of the two samples, as outlined in [51].

(i) If

(5) $$\frac{S_{1}^{2}}{S_{2}^{2}}\in \left[\frac{1}{3};3\right]$$

Then, the variances $S_{1}^{2}$ and $S_{2}^{2}$ of the two samples can be considered approximately equal, and the *t*-test with equal variance (pooled *t*-test) is applied.

The relation determines the statistical term *t*:

(6) $$t=\;\frac{X_{M1}-X_{M2}}{S_{p}\sqrt{\frac{1}{n_{1}}\;+\;\frac{1}{n_{2}}}}$$

where *X_M_*_1_ and *X_M2_* are the means of the analysed samples, n_1_ and n_2_ are the sizes of the two samples, and $s_{p}$ is the combined standard deviation, calculated as follows:

(7) $$s_{p}=\;\sqrt{\frac{\left(n_{1}-1\right)s_{1}^{2}\;+\;\left(n_{2}\;-\;1\right)s_{2}^{2}}{n_{1}\;+\;n_{2}\;-\;2}}$$

where $s_{1}$ and $s_{2}$ are the standard deviations of the two analysed samples.

The degrees of freedom are determined as

(8) $$d_{f}=n_{1}+n_{1}-2$$

(ii) If the variances of the samples being compared differ significantly, the Welch test is utilized, in which the statistical term *t* is calculated using the following relation:

(9) $$t=\;\frac{X_{M1}-X_{M2}}{\sqrt{\frac{s_{1}}{n_{1}}+\frac{s_{2}}{n_{2}}}}\;$$

In this case, the degrees of freedom are approximate, according to the Welch–Satterthwaite equation:

(10) $$d_{f}\approx \frac{{\left(\frac{s_{1}^{2}}{n_{1}}+\frac{s_{2}^{2}}{n_{2}}\right)}^{2}}{\frac{{\left(\frac{s_{1}^{2}}{n_{1}}\right)}^{2}}{n_{1}-1}+\frac{{\left(\frac{s_{2}^{2}}{n_{2}}\right)}^{2}}{n_{2}-1}}\;$$

In both variants, (i) and (ii), the *p*-value related to the statistical term *t* is calculated. This *p*-value indicates the probability of observing a difference between the sample means that is at least as extreme as the one observed, assuming the null hypothesis is true [52].

For a two-sided test, the *p*-value is

(11) $$p=2\;P(T\geq \left|t\right|)$$

In this context, *t* represents the value of the previously calculated *t*-statistic, *T* is the random variable that follows the Student’s *t*-distribution with a specific number of degrees of freedom ($d_{f}$), and *P* denotes the chosen confidence level.

To determine the *p*-value, we considered a significance level of α = 0.05, which corresponds to a 95% confidence level. When conducting multiple statistical comparisons—in this case, comparing two sets of samples from a total of *m* = 3 samples—the risk of error increases. Therefore, to control the overall error rate, the Bonferroni correction was applied [53]. This adjustment modifies the significance threshold α to an adjusted level, denoted as α_aj_. This ensures that we maintain the integrity of our statistical conclusions:

(12) $$\alpha_{aj}=\;\frac{\alpha }{m}$$

Each test was assessed using a more stringent threshold, α_aj_ = 0.017. The evaluation criteria were as follows:

- If *p* < α_aj_, the null hypothesis is rejected, indicating that the differences between the two samples are statistically significant.
- If *p* ≥ α_aj_, there is insufficient evidence to reject the null hypothesis, meaning the differences between the two samples are considered statistically insignificant.

Additionally, the statistical analysis included an evaluation of effect size by calculating Cohen’s *d*. This metric expresses the difference between the means of the two samples in relation to their combined standard deviation [54]:

(13) $$d=\;\frac{X_{M1}-X_{M2}}{S_{p}}$$

The differences between the two samples were represented as the percentage difference (Δ%), which quantifies the relative variation between the means being compared [55]:

(14) $$\Delta \%=\;\frac{\left|X_{m1}-X_{m2}\right|}{min(X_{m1},X_{m2})}\cdot 100$$

The statistical procedure employed in this analysis utilized the *t*-test for two independent samples, complemented by *p*-value analysis and Bonferroni correction. Additionally, the effect size was determined using Cohen’s *d*, along with the percentage difference (Δ%). This approach ensures a rigorous evaluation of both the statistical significance and practical relevance of the differences observed in the quality parameters of the parts produced with the tools under investigation.

## 3. Results

### 3.1. SPIF Microchips: Visual Appearance of Formed Surfaces

Incremental plastic deformation forming at a single point generates microchips due to intense local friction between the active area of the tool and the workpiece. The size of these microchips depends on specific forming conditions, which are influenced by factors such as the shape of the tool’s active area, the material of the workpiece, the geometric shape of the part, the feed rate (v), the tool speed (n), and the type of lubricant used. Consequently, after each experiment, the microchips produced during forming were visually analyzed. They were then collected and examined under an optical microscope.

Depending on the functional role of thin sheet metal parts, such as exterior body parts in the automotive industry, the visual appearance of the surface can be a parameter that defines the quality of the part. Therefore, after the microchips were analyzed, the parts were cleaned and visually inspected.

#### 3.1.1. Microchips and Visual Appearance of Surfaces When Forming with the SS10 Tool

**Figure 15:**
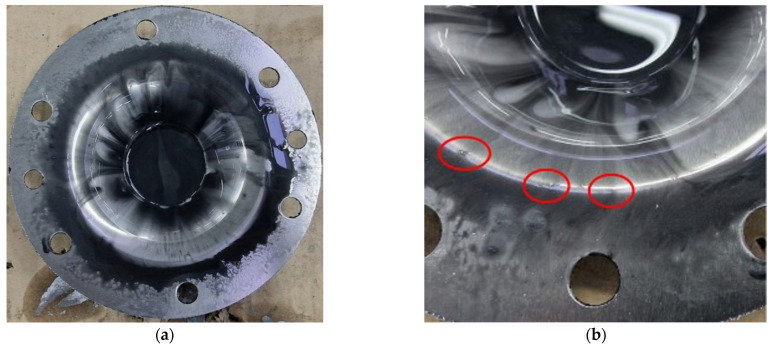
Macroscopic appearance of the workpiece formed with a 10 mm radius hemispherical head tool (SS10), immediately after the process is completed: (**a**) overall image; (**b**) detail with microchips (in the red circles).

Macroscopic images of the parts formed with a tool featuring a hemispherical head and a radius of 10 mm (designated as SS10) are presented in Figure 15. It is observed that the surface of the part has a layer of very fine microchips. These microchips have detached from the workpiece due to the friction between the tool and the blank during the SPIF process.

**Figure 16:**
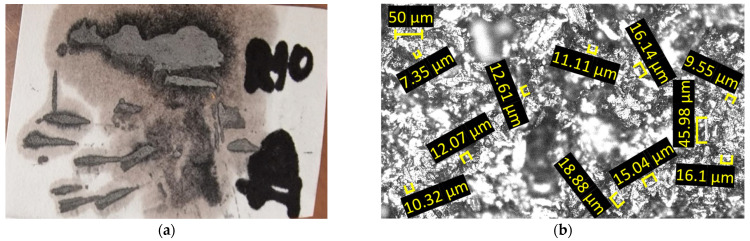
Optical analysis of microchips detached during forming with SS10: (**a**) sampled microchips; (**b**) microscopic image of microchips.

The microchips were analyzed optically, with a 50× magnification, and the images of them are presented in Figure 16. The dimensions of the microchips range from 7 to 18 μm, with the predominant dimension being approximately 12 μm.

**Figure 17:**
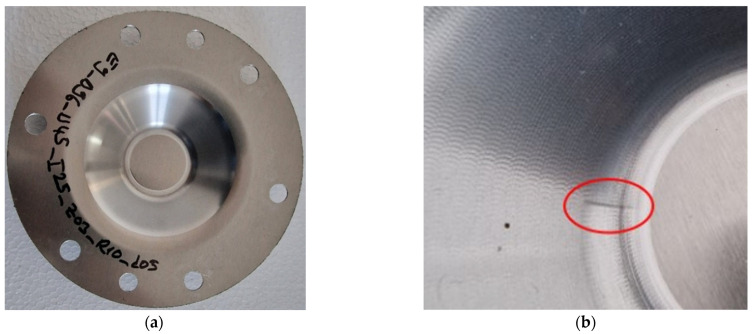
Visual appearance of the part formed with the SS10 tool, after cleaning it: (**a**) overall image; (**b**) detail with the trace left by the tool at the end of the process (in the red circle).

Images that highlight the visual appearance of the part made with the SS10 tool, after cleaning it of microchips and lubricant, are presented in Figure 17. The inner surface of the truncated cone has a macroscopic appearance of good quality, with no major scratches visually observable. At the tip of the truncated cone, the one made last in the forming process, a trace imprinted in the piece is noticeable (see Figure 17b), which is created by the tool reaching the end of its trajectory at the moment of stopping the process.

**Figure 18:**
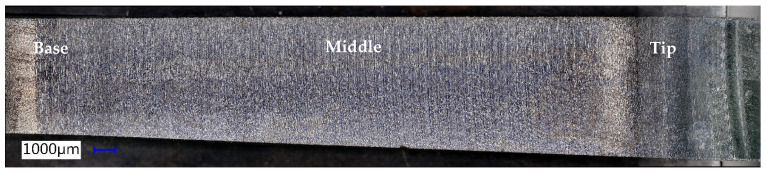
Detail of the part surface formed with the SS10 tool.

The good quality aspect of the formed surface also results from the detail in Figure 18. This image highlights the traces printed on the tool part, determined by the tool trajectory, the shape of its active area, and the incremental step, Δz, traces that are specific to the SPIF process.

#### 3.1.2. Microchips and Visual Appearance of Surfaces When Forming with the SS5 Tool

**Figure 19:**
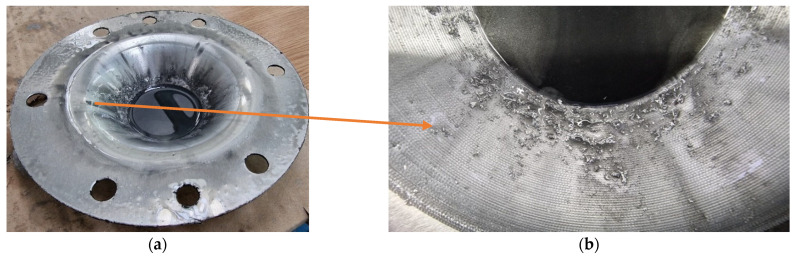
Macroscopic appearance of the part formed with a 5 mm radius hemispherical head tool (SS5), immediately after the process was completed: (**a**) overall view; (**b**) detail with microchips.

The images displaying the macroscopic appearance of the parts formed using a tool with a hemispherical head and a radius of 5 mm (SS5) are shown in Figure 19. Immediately after the SPIF process, it is observed that the formed surface of the part contains a significant volume of microchips, many of which are relatively large in size.

SPIF forming with a hemispherical head punch and a radius of 5 mm generates a significant amount of visible, large chips during the deformation process. These chips are clearly shown in Figure 19b, which was taken after lubricant oil leaked onto the bottom of the part. The presence of these chips indicates a considerable level of abrasion between the tool and the workpiece, resulting in a high volume of chips. This accumulation has a detrimental effect on the quality of the inner surface of the finished part.

**Figure 20:**
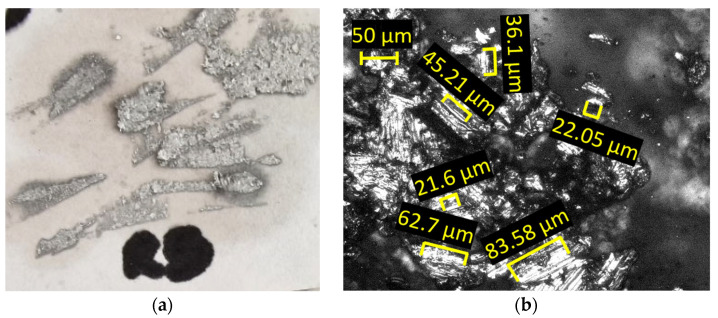
Optical analysis of microchips detached during forming with the SS5 tool: (**a**) sampled microchips; (**b**) macroscopy with microchips.

The optical analysis of the microchips, conducted at 50× magnification, as displayed in Figure 20, shows dimensions ranging from 21 to 83 µm, with the most common measurement around 45 µm.

**Figure 21:**
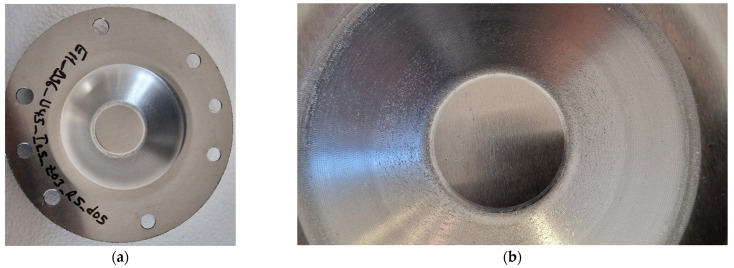
The visual appearance of the part formed with the SSR5 tool after cleaning: (**a**) overall view; (**b**) view of the formed surface.

The macroscopic appearance of the part formed with the SS5 tool, after cleaning, is shown in Figure 21. The inner surface of the truncated cone exhibits a low-quality appearance, with numerous scratches visibly present on the formed surface.

During the forming process with the SS5 tool, a significant number of chips of varying sizes are produced as the part undergoes incremental deformation. These chips begin to form at the start of the SPIF process and increase in quantity as the tool progresses along the generation path. The use of lubricant causes the chips to coagulate, resulting in an abrasive mixture that infiltrates the contact area between the punch and the blank. This can lead to damage to the formed surface. Larger chips can cause scratches and leave noticeable marks, as illustrated in Figure 21b.

**Figure 22:**
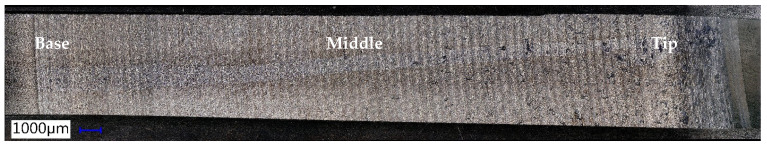
Detail of the part surface formed with the SS5 tool.

The geometry of the objective part has a significant impact on the SPIF process. As the process advances, the lubricant tends to flow towards the tip of the truncated cone. Consequently, a larger accumulation of chips occurs at the tip, accentuating the abrasion phenomenon. This results in a reduced number of scratches at the base of the truncated cone (the larger diameter), while the tip exhibits a much higher concentration of scratches. The poor quality of the formed surface is further illustrated in Figure 22. In this image, the traces left on the tool part are visible, which are typical of the SPIF process, along with the microchips remaining on the formed surface. Notably, the size of these microchips increases towards the tip of the cone.

#### 3.1.3. Microchips and Visual Appearance of Surfaces When Forming with the ETVR Tool

**Figure 23:**
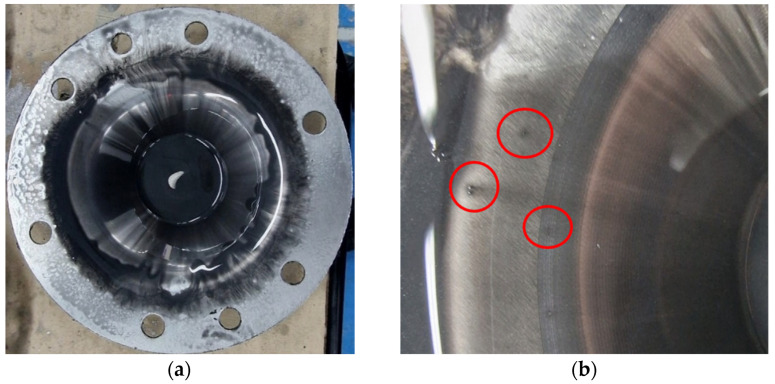
Macroscopic appearance of the part formed with the ETVR variable radius eccentric tool, immediately after the process was completed: (**a**) overall view; (**b**) detail with microchips (in the red circle).

**Figure 24:**
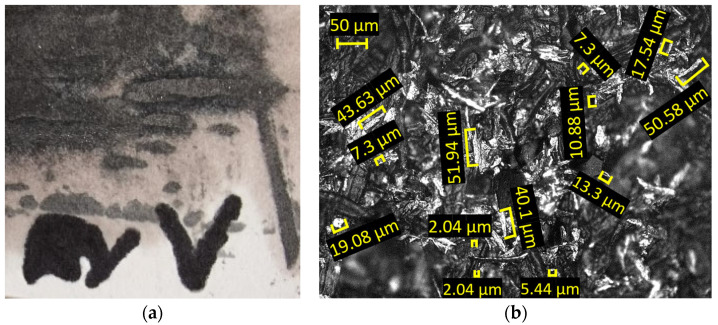
Optical analysis of microchips detached during forming with the ETVR tool: (**a**) sampled microchips; (**b**) macroscopy with microchips.

The chips produced during forming with the innovative ETVR tool are minimal in quantity, as shown in Figure 23. They are exceptionally fine, as illustrated in Figure 24a. An analysis conducted under an optical microscope with a 50× magnification, as depicted in Figure 24b, reveals that the chip dimensions range from 2 to 51 µm, with the most common size being approximately 8 µm.

**Figure 25:**
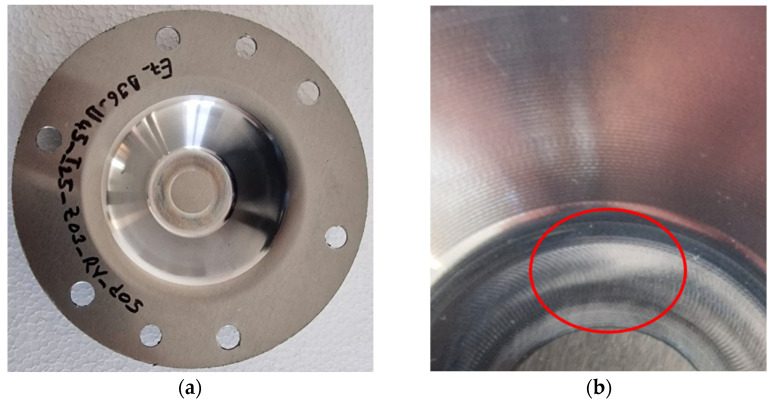
The visual appearance of the part formed with the ETVR tool after cleaning: (**a**) overall view; (**b**) view of the formed surface at the tip of the truncated cone, highlighting the large flat area (in the red circle).

The visual appearance of the part formed with the ETVR tool, after its cleaning, is shown in Figure 25. The inner surface of the truncated cone has a macroscopic appearance of very good quality, with no major scratches visually observed. At the tip of the truncated cone, a larger flat area is noticeable (Figure 25b), which is created by the front flat area of the active part of the ETVR tool. This has the favorable effect of reducing the cushion effect that occurs at the tip of the truncated cone, a phenomenon specific to the SPIF process [22,27,28,50].

**Figure 26:**
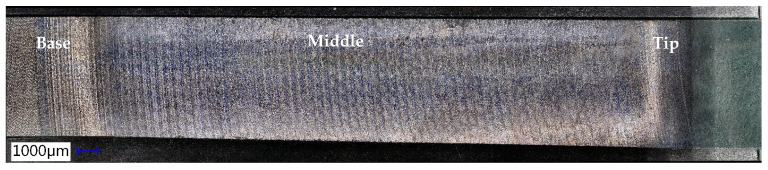
Detail of the part surface formed with the ETVR tool.

The high-quality appearance of the SPIF-processed surface is illustrated in Figure 26. The surface of the part does not exhibit scratches from chips; only the tool’s imprint marks are visible.

### 3.2. Surface Roughness of Parts Formed by SPIF

#### 3.2.1. Surface Roughness of the Blank

The statistical values of the roughness parameters Ra and Rt, obtained by measuring the roughness of the blank using Equations (1) to (4), are summarized in Table 5 and Table 6.

The relative standard deviation (RSD) indicator values for the parameter Ra, which are below 4.5% for both roughness measurement directions, indicate good repeatability of these measurements. The high relative standard deviation (RSD) values, exceeding 25%, for the Rt parameter in the rolling direction indicate a considerable non-uniformity in the sheet surface. The rolling texture of the sheet influences this non-uniformity, highlighting the sensitivity of this parameter to local defects.

The significant differences between the average values of the two roughness parameters, Ra and Rt, in the rolling direction (RD) and the transverse direction (TD), indicate anisotropy in the roughness based on the measurement direction. This anisotropy is attributed to the sheet production method, specifically the rolling process.

#### 3.2.2. Surface Roughness of Parts Formed with the SS10 Tool

**Figure 27:**
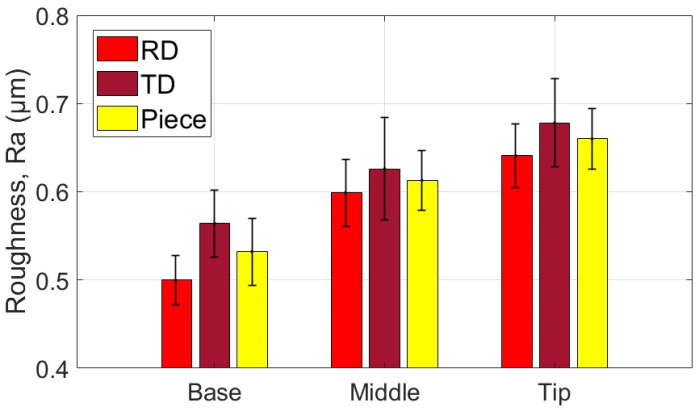
The roughness parameter Ra of the piece formed by SPIF with the SS10 tool.

**Figure 28:**
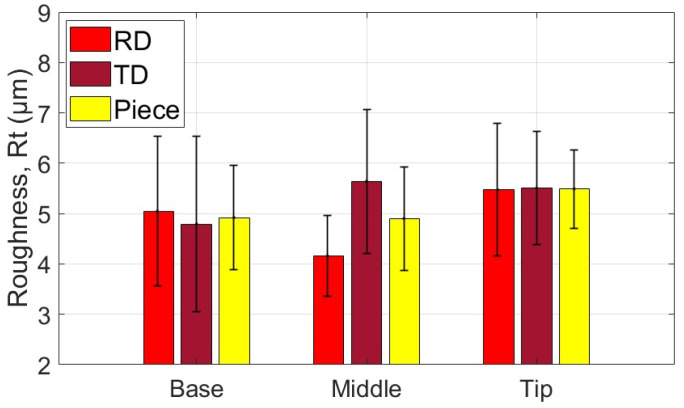
The roughness parameter Rt of the piece formed by SPIF with the SS10 tool.

The average values of the roughness parameters Ra and Rt, along with the associated statistical quantities, are presented for the measurement areas and directions of the part formed with the SS10 tool in Table 7 and Table 8. Additionally, the average values of parameters Ra and Rt related to the two specific directions of the workpiece and the part are illustrated graphically in Figure 27 and Figure 28.

To examine the presence of “local accidents” on the surface of the formed part, we calculated the ratio between Rt and Ra in the areas where roughness was measured. The values of this ratio are shown in Table 9.

Based on the analysis of the data presented in Table 7, Table 8 and Table 9 and the illustrations in Figure 27 and Figure 28, the following points can be highlighted:

- The average values of the roughness parameters Ra and Rt on the formed part are higher than those of the workpiece in both analyzed directions, RD and TD. However, the roughness of the formed part is more consistent than that of the workpiece. This difference can be attributed to the SPIF process, which alters the surface asperities due to the direct contact between the tool and the workpiece. The increase in Ra indicates a specific texture created by the tool’s path. At the same time, the rise in Rt is the result of indentations caused by contact pressure, as well as isolated occurrences such as scratches, microadhesion, and microchips that reach the contact area between the tool and the workpiece.
- The average values of the Ra parameter in the RD (rolling direction) are lower than those in the TD (transverse direction). The difference is more pronounced at the base of the cone, measuring approximately 0.064 μm, and smaller at the midpoint, around 0.027 μm. In contrast, the average values of the Rt parameter vary between the two directions; they are greater in the RD direction at the base of the cone, larger in the TD direction at the midpoint, and nearly equal at the tip of the cone. This variation suggests a stabilization of isolated events specific to the SPIF process.
- The evolution of the Ra parameter along the cone generator is similar in both the RD and TD directions: it shows lower values at the base and higher values at the tip. In contrast, the evolution of the Rt parameter differs between the two directions; however, the highest values are consistently found at the tip of the cone. This behavior is influenced by the forming process, during which more microchips accumulate at the tip of the forming cone. These microchips also reach the contact area between the tool and the workpiece, leading to damage to the formed surface. The variations in the Rt parameter are less pronounced in the TD direction. This is because the pressure exerted by the tool and its friction with the workpiece tend to align the surface asperities in the direction of the tool’s movement, resulting in more uniform and relatively smooth traces in that direction.
- The low values of the relative standard deviation (RSD < 6.383%) for the Ra parameter measured in the specific areas of interest (B, M, T) indicate that the measurements have good repeatability. In contrast, the higher relative standard deviation values for the Rt parameter (RSD = 14% to 26%) are expected, as this parameter is sensitive to extremes. Occasional extremes, such as scratches and asperities, can increase the dispersion of the values.
- The Rt/Ra ratio shows the highest values at the base of the cone in the RD direction (10.098), while the lowest values are found in the middle of the cone (6.947), also in the RD direction. The elevated values at the cone’s base can be attributed to the lower Ra parameter and the presence of microbreaks and vibrations in the material, which are typical during the initial stages of forming. In the TD direction, the values of this ratio fall within a narrower range (8.121 to 9.011) due to the smaller variation of the Rt parameter in that direction.

#### 3.2.3. Surface Roughness of Parts Formed with the SS5 Tool

**Figure 29:**
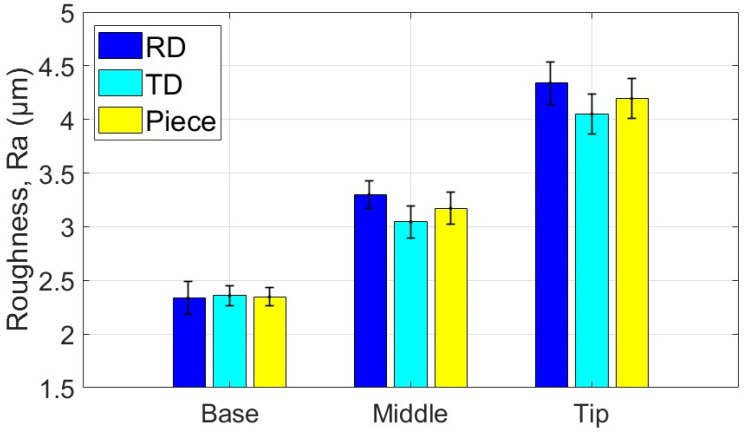
The roughness parameter Ra of the piece formed by SPIF with the SS5 tool.

**Figure 30:**
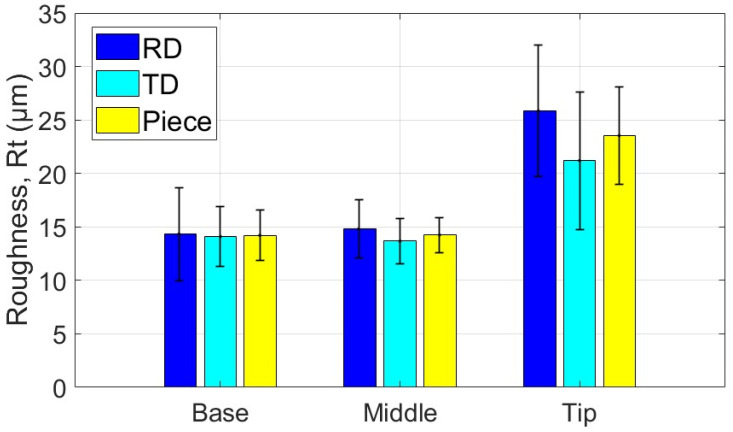
The roughness parameter Rt of the piece formed by SPIF with the SS5 tool.

The average values of the roughness parameters Ra and Rt, along with the associated statistical quantities, are presented for the measurement areas and directions of the part formed with the SS5 tool in Table 10 and Table 11. Additionally, the average values of parameters Ra and Rt related to the two specific directions of the workpiece and the part are illustrated graphically in Figure 29 and Figure 30.

To examine the presence of “local accidents” on the surface of the formed part, we calculated the ratio between Rt and Ra in the areas where roughness was measured. The values of this ratio are shown in Table 12.

Based on the analysis of the data in Table 10, Table 11 and Table 12 and the illustrations in Figure 28 and Figure 30, several key points can be highlighted:

- The average roughness parameters Ra and Rt on the formed part are significantly higher than those of the blank in both analyzed directions: RD (radial direction) and TD (tangential direction). Specifically, the differences exceed 2.2 µm for the Ra parameter in the RD direction and over 1.9 µm in the TD direction. For the Rt parameter, the differences are even more pronounced, with increases of over 12.592 µm in the RD direction and over 11.181 µm in the TD direction. These substantial increases in roughness result from the limited contact area between the tool and the workpiece, leading to severe local plastic deformation and microchipping. Consequently, the surface asperities on the formed part are more pronounced, showing more accentuated tool marks and deeper scratches due to the abrasive contact with the tool.
- As the deformation process advances, the average values of the Ra and Rt parameters increase more significantly in the TD (transverse direction) compared to the RD (rolling direction). The maximum differences are observed at the tip of the cone, with Ra measuring 0.29 µm and Rt measuring 4.467 µm. This change is attributed to the very high contact pressure of the tool over a limited contact area, which leads to a deterioration of the initial texture of the blank. Consequently, the roughness in the RD increases at a greater rate than that in the TD.
- A critical increase in the values of the Ra and Rt parameters is observed from the base of the cone towards its tip, which is determined by the intensification of the deformation process and the accumulation of microchips in the toolworkpiece contact area. In the case of the Ra parameter, the increase is relatively uniform. In contrast, the increase in the Rt parameter occurs in the tip area of the cone, primarily due to the accumulation of large microchips in significant quantities towards the tip. Their penetration into the toolworkpiece contact area produces a large number of scratches on the part, which are much more visible.
- The low values of the relative standard deviation (RSD < 4.68%) for the locally measured Ra parameter in the areas of interest (B, M, V) indicate good repeatability of the measurements. In contrast, the higher relative standard deviation values for the Rt parameter (RSD = 10.9% to 21.3%) are understandable, as the Rt parameter is highly sensitive to extreme values.
- The Rt/Ra ratio exhibits higher values at the base of the cone and lower values in its middle section, in both the RD and TD directions. The maximum value recorded is 6.138 in the RD direction, while the minimum is 4.489, also in the RD direction. This variation in the Rt/Ra ratio can be attributed to the local high pressure exerted by the tool at the base of the cone, which hampers lubrication and leads to the formation of deep scratches. As a result, the Ra value increases moderately, while the Rt value rises significantly due to these scratches. In the middle section of the cone, the tool operates under more favorable conditions, resulting in better sliding and more uniform material deformation. Consequently, the Ra value increases (indicating a rougher surface), but the Rt value remains relatively unchanged. At the tip of the cone, working conditions deteriorate mainly due to the accumulation of microchips. The larger size of these chips results in deeper scratches, which causes an increase in the Rt parameter.

#### 3.2.4. Surface Roughness of Parts Formed with the ETVR Tool

**Figure 31:**
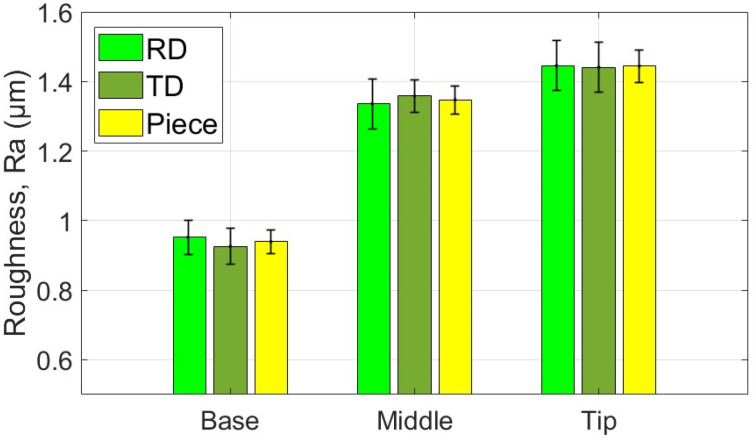
The roughness parameter Ra of the piece formed by SPIF with the ETVR tool.

**Figure 32:**
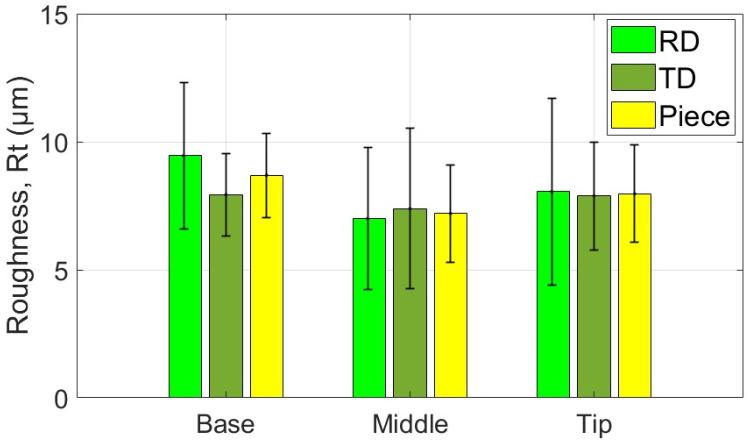
The roughness parameter Rt of the piece formed by SPIF with the ETVR tool.

The average values of the roughness parameters Ra and Rt, along with the associated statistical quantities, are presented for the measurement areas and directions of the part formed with the ETVR tool in Table 13 and Table 14. Additionally, the average values of parameters Ra and Rt related to the two specific directions of the workpiece and the part are illustrated graphically in Figure 31 and Figure 32.

To assess the presence of “local accidents” on the surface of the formed part, we calculated the ratio of Rt to Ra in the areas where roughness measurements were taken. The values of this ratio are presented in Table 15.

Analysis of the data presented in Table 13, Table 14 and Table 15, along with the graphical representations in Figure 31 and Figure 32, reveals several important points:

- The average roughness parameters, Ra and Rt, of the formed part are higher than those of the blank in both the radial direction (RD) and tangential direction (TD). However, the roughness values of the formed part are more consistent compared to those of the blank, similar to the results obtained when using the SS10 tool. Specifically, the increases in the roughness parameters compared to the blank are more than 0.837 µm in the RD direction and over 0.472 µm in the TD direction for the Ra parameter. For the Rt parameter, the increases are greater than 5.622 µm in the RD direction and over 4.901 µm in the TD direction. These increases are larger than those observed with the SS10 tool, but significantly lower than those recorded with the SS5 tool.
- The average values of the parameters Ra and Rt in the radial direction (RD) are higher than those in the transverse direction (TD) at both the base and the tip of the cone. The difference is more significant at the base, with Ra showing a difference of 0.66 μm and Rt showing a difference of 1.523 μm. However, in the middle of the cone, both roughness parameters are greater in the TD direction than in the RD direction, although the differences are relatively slight: 0.022 μm for Ra and 0.386 μm for Rt.
- The evolution of the Ra parameter along the cone generator is similar to that from forming with the SS10 and SS5 tools, in both RD and TD directions: there are lower values at the base and higher values towards the tip. The increases in the Ra parameter values are monotonous and of the same intensity in the two directions, of 0.494 μm in the RD direction and 0.515 μm in the TD direction. In contrast, the evolution of the Rt parameter is different from that of forming with the other two tools, decreasing from the base of the cone towards its tip, in both directions, and with minimum values at its middle. This evolution, in which the higher values of Rt are higher at the base of the cone, can be explained as follows: at the beginning of the formation, the lubricant film can break locally, and the shape of the tool can favor this, through intermittent contact. Thus, the tool can generate extreme events, such as scratches and grooves, which result in higher values of Rt. The deformation process is better towards the middle area, when lubrication becomes better, and the number of microchips is not too high. It gets slightly worse towards the tip of the cone, with the increase in the number of microchips.
- The low values of the relative standard deviation (RSD < 3.9%) for the Ra parameter measured locally in the areas of interest (B, M, V) indicate good repeatability of the measurements. In contrast, the higher relative standard deviation values for the Rt parameter (RSD = 14.019% to 31.59%) are still considered reasonable. This is because the Rt parameter is highly sensitive to extreme values.
- The evolution of the Rt/Ra ratio values is similar to that observed when using hemispherical head tools. The highest Rt/Ra ratio is found at the base of the cone in the RD direction, measuring 9.932, while the lowest value occurs in the middle of the cone, at 5.242, also in the RD direction. The Rt/Ra ratio values from this method are slightly lower than those obtained using the SS10 tool, but higher than those achieved with the SS5 tool.

### 3.3. Microhardness of Parts Processed by SPIF

#### 3.3.1. Microhardness of the Blank

By measuring the HV microhardness values on the blank, we calculated the average HV parameter and the associated statistical quantities in the analyzed areas, using Relations (1) to (4). These results are shown in Table 16.

The relative standard deviation (RSD) indicator is very low, remaining below 1.4% for both directions of microhardness measurement. This indicates good repeatability of the measurements. Furthermore, the low margin of error values in each measurement direction demonstrate a high level of homogeneity within the material. However, there is a significant difference between the average values in the rolling direction (RD) and the transverse direction (TD). The higher RSD values and limit errors suggest that the blank exhibits anisotropy in the two analyzed directions. This anisotropy is likely a result of the rolling method used to produce the sheet.

#### 3.3.2. Microhardness of the Surface Layer of Parts Formed with the SS10 Tool

**Figure 33:**
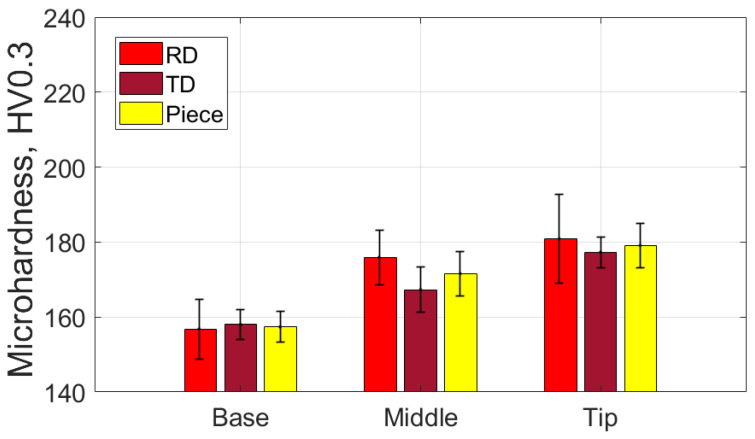
The average microhardness (HV 0.3) of the piece formed by SPIF with the SS10 tool.

Table 17 presents the average values of the HV parameter, along with the statistical quantities associated with it, based on measurements in the specific areas and directions for the part formed with the SS10 tool. Figure 33 graphically displays the average HV microhardness values corresponding to the two directions particular to the workpiece and the part itself.

Based on the analysis of the data presented in Table 17 and the graphical representations in Figure 33, the following observations can be made:

- The average microhardness values (HV 0.3) of the formed part are generally higher than those of the blank in both analyzed directions: radial (RD) and transverse (TD), as well as across the entire part. Near the base of the cone, the microhardness values are similar to those of the blank; however, they increase toward the tip of the cone in both directions (RD and TD). This indicates that the material of the formed part exhibits properties similar to the blank at the base, where the plastic deformation process begins, and becomes progressively harder toward the tip.
- The average microhardness values (HV 0.3) in the TD direction are lower than those in the RD direction at both the middle and the tip of the cone. The maximum difference occurs at the middle of the cone’s generator, measuring approximately 9 HV 0.3. This suggests that, while the material’s anisotropy remains present, it is slightly reduced compared to the initial state of the workpiece in some areas of the generatrix cone.
- The low relative standard deviation values (RDS < 4.6%) indicate that the measurements taken across all analyzed areas and in both directions show good repeatability.

#### 3.3.3. Microhardness of the Surface Layer of Parts Formed with the SS5 Tool

**Figure 34:**
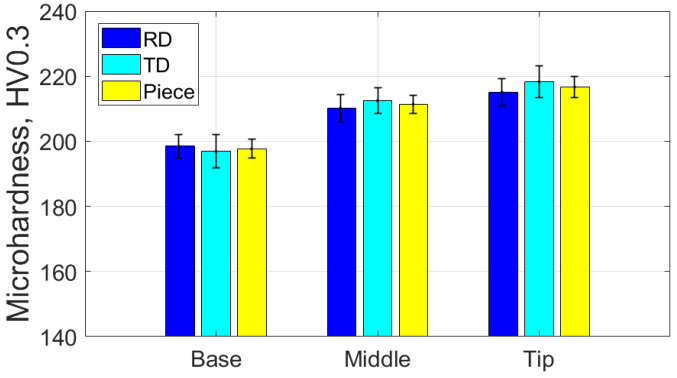
The average microhardness (HV 0.3) of the piece formed by the SPIF with the SS5 tool.

Table 18 shows the average values of the HV parameter, along with the related statistical quantities, based on measurements taken in specific areas and directions for the part formed using the SS5 tool. Figure 34 provides a graphical representation of the average HV microhardness values corresponding to two specific directions of the workpiece and the part itself.

From the analysis of the data in Table 18 and the graphical representations in Figure 34, the following observations can be made:

- The average microhardness values (HV0.3) of the formed parts are significantly higher than those of the blank, both in the radial direction (RD) and the transverse direction (TD) across the investigated areas (B, M, T). On average, microhardness increases by at least 38 HV0.3 in the RD and 53 HV0.3 in the TD. These increases indicate that the material of the formed part has undergone significant hardening due to the localized intensity of the plastic deformation process.
- The differences in average microhardness values (HV0.3) between the RD and TD directions are minimal. At the base of the cone, the difference is 1.6 HV0.3, with higher values in the RD direction. In the middle of the cone, the difference is 2.3 HV0.3, with higher values in the TD direction. At the tip of the cone, the difference is 3.2 HV0.3, again with higher values in the TD direction. This suggests that the material’s anisotropy has been reduced compared to the initial state of the blank.
- The average microhardness values (HV0.3) along the generator of the cone increase from the base to the tip, in both the RD and TD directions. This trend indicates that the hardening process intensifies as singlepass incremental forming (SPIF) progresses.
- The very low relative standard deviation (RSD < 2.1%) reflects a high level of repeatability for the measurements taken in all analyzed areas and in both directions.

#### 3.3.4. Microhardness of the Surface Layer of Parts Formed with the ETVR Tool

**Figure 35:**
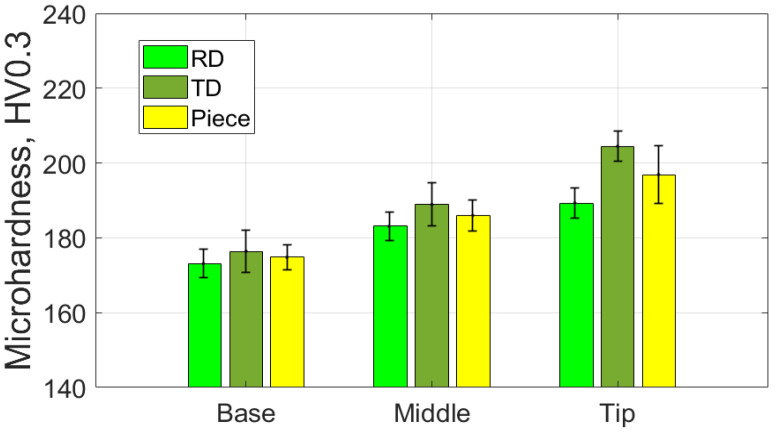
The average microhardness (HV 0.3) of the piece formed by the SPIF with the ETVR tool.

Table 19 presents the average values of the HV parameter, along with relevant statistical information, based on measurements taken in specific areas and directions of the part formed with the ETVR tool. Figure 35 illustrates a graphical representation of the average HV microhardness values for two particular directions of the workpiece and the part itself.

Analyzing the data from Table 19 and the visuals in Figure 35 reveals the following observations:

- The average microhardness values (HV0.3) of the formed parts are consistently higher than those of the blank in both analyzed directions: Radial Direction (RD) and Tangential Direction (TD) across all zones of the cone generator (Base, Mid, Tip). On average, the microhardness increases by at least 14 HV0.3 for RD and 18 HV0.3 for TD. These increases demonstrate that the material of the formed part has hardened in all areas.
- The differences in average microhardness values between the RD and TD directions become more pronounced from the base to the tip of the cone. Specifically, the differences are 3.3 HV0.3 at the base, 5.8 HV0.3 in the middle, and 15.2 HV0.3 at the tip, with consistently higher values found in the TD direction. This indicates a significant change in the material’s anisotropy compared to the original state of the blank, particularly demonstrating greater microhardness in the TD direction towards the tip of the cone.
- Additionally, the average HV0.3 microhardness values along the cone generator increase from the base to the tip in both analyzed directions (RD and TD), suggesting that the hardening process intensifies as forming progresses.
- Finally, the very low relative standard deviation (RSD < 4.2%) reflects a high level of repeatability in the measurements taken across all analyzed areas and in both directions.

## 4. Discussions

The results presented above, evaluated independently of the tools used, emphasize the impact of tool geometry on the quality parameters of surfaces produced by SPIF. In this section, we conduct a comparative analysis of the results obtained from three different tools. We will also provide a statistical comparison of the surface quality parameters and examine how the anisotropy of the blank affects the results.

### 4.1. Analysis and Interpretation of Surface Roughness: A Comparative Study

The experimental results showed a progressive increase in surface roughness on the truncated cone generator, from the base to the tip, for all parts formed with the three tools. This trend can be attributed to the formation and accumulation of microchips in the contact area between the tool and the workpiece, particularly towards the end of the forming process at the tip of the cone. The formation and accumulation of microchips were observed with all three tools, but the intensity varied, as presented in Table 20:

- The SS5 tool generated the most significant and most numerous microchips, measuring up to 83 μm. This larger chip size was associated with higher roughness values and a poorer visual appearance of the formed surface.
- The SS10 tool produced significantly finer microchips, ranging from 7 to 18 μm, which resulted in a visually superior surface.
- The ETVR tool generated the fewest microchips, predominantly of a fine size (8 μm), leading to a uniform aesthetic appearance of the formed surface and lower roughness.

**Figure 36:**
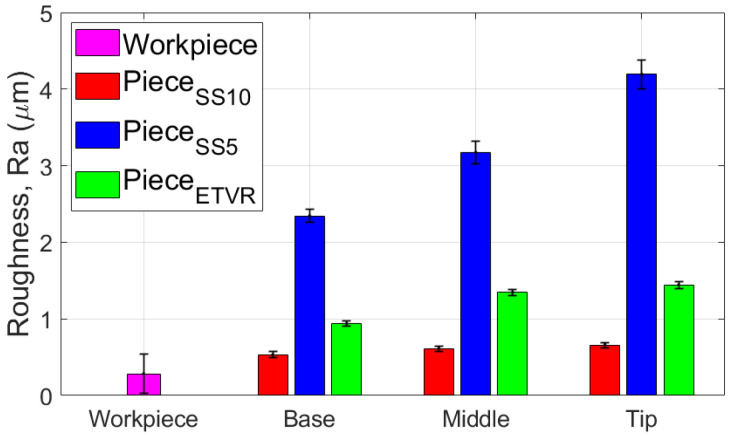
The roughness parameter Ra of the parts formed with the three different tools.

The experimental results indicated a progressive increase in the roughness parameter Ra along the cone truncated generator, from the base to the tip, across all parts formed with the three tools, as illustrated in Figure 36. The degree of this increase varied depending on the tool geometry:

- The SS5 tool, which has the smallest radius, produced the highest roughness values, reaching up to 4.4 μm at the tip. It also exhibited the most significant variation in roughness between the base and the tip, measuring 1.84 μm.
- The SS10 tool, featuring the largest radius, resulted in significantly lower roughness values, around 0.6 μm at the tip, and showed the most minor variation along the cone generator, only 0.13 μm.
- The innovative ETVR tool, designed with an eccentric shape and variable radius, yielded intermediate results, with average roughness Ra values ranging from 0.94 to 1.44 μm. It also provided a uniform visual appearance of the surface.

Figure 36 illustrates the impact of the tool’s radius on the roughness parameter Ra of the formed surface. The SS5 tool, featuring the smallest radius, resulted in the highest roughness values—up to 4.4 μm at the tip—and exhibited the most significant variations between the base and the tip (1.84 μm). In contrast, the SS10 tool, with the largest radius, produced a significantly lower roughness of approximately 0.6 μm and the smallest variations along the cone generator (0.13 μm). The innovative ETVR tool, which features an eccentric shape and a variable radius, yielded intermediate results, achieving an average roughness Ra of between 0.94 and 1.44 μm; however, it provided the best visual appearance in terms of surface uniformity.

**Figure 37:**
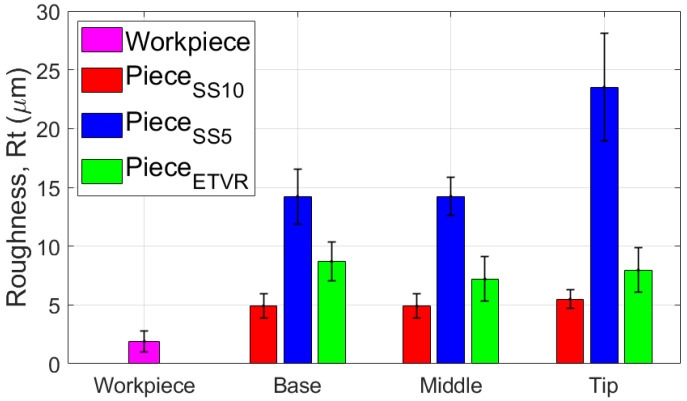
The roughness parameter Rt of the parts formed with the three different tools.

Also, the results regarding the roughness parameter Rt show significant differences between the parts formed with the three tools used, as illustrated in Figure 37:

- The SS5 tool produced the highest Rt values, exceeding 25 μm in the tip area. This reflects the deep scratches and grooves caused by the abrasive contact between the tool and the workpiece, along with the accumulation of microchips.
- The SS10 tool yielded moderate Rt values, approximately 5–5.5 μm. The measurements fluctuated across different areas, with a slight increase in Rt at the tip due to the buildup of microchips.
- The innovative ETVR tool achieved intermediate Rt results, with an average value ranging from 7 to 9 μm. There was a decreasing trend from the base to the middle of the tool, followed by a slight increase at the tip.

To statistically compare the roughness parameters Ra and Rt of the formed parts using different tools, a statistical analysis of the results was conducted based on the procedure outlined in Section 2 (relations 5–14). The statistical analysis results are shown in Table 21 for the Ra parameter and Table 22 for the Rt parameter.

The analysis of the results presented in Table 21 and Table 22 demonstrates that the differences in the average values of the Ra and Rt parameters for the parts formed with the three tools are highly statistically significant (*p* << 0.001) across all examined areas (Base, Middle, Top). The following points are noteworthy regarding the comparisons made:

- The differences in roughness parameters between the SS10 and SS5 surfaces are statistically significant and substantial. The Cohen’s *d* values for these comparisons are extremely high, exceeding 21 for the Ra parameter and over 4.8 for the Rt parameter (in absolute terms). Additionally, the percentage effects are also considerable, exceeding 188%. This indicates that the tools being compared yield vastly different performances in terms of workpiece roughness. Specifically, the SS5 tool results in Ra values of the formed surface that are 4 to 6 times higher than those produced by the SS10 tool. Moreover, the SS5 tool produces Rt values greater than 25 µm, which is significantly above the approximately 5 µm values achieved by the SS10 tool.
- The comparison between SS5 and ETVR reveals significant and substantial differences in the roughness parameters of the two surfaces. Although the Cohen’s *d* values are somewhat lower than in the previous comparison, they are still notable, exceeding 15 for the Ra parameter and over 2.5 for the Rt parameter (in absolute values). Additionally, the percent change effects are pretty significant, surpassing 63%. The two tools being compared demonstrate different performances regarding part roughness; however, the ETVR tool is not as effective as the SS10.
- The comparison between ETVR and SS10 reveals that the differences in roughness parameters of the two surfaces are both statistically significant and substantial. The Cohen’s *d* values are the lowest of all comparisons made, with a value exceeding 10 for the Ra parameter and exceeding 1.5 for the Rt parameter (in absolute terms). While the percentage effects are smaller than in previous comparisons, they still exceed 45%. The two tools being compared exhibit different performances in terms of workpiece roughness for both roughness parameters.

As can be seen from Figure 36 and Figure 37, but also from the statistical evaluation, there are significant differences, evident from a technological point of view, between the roughness of the piece formed by the SS5 tool and the roughness of the pieces formed with the other two tools.

**Figure 38:**
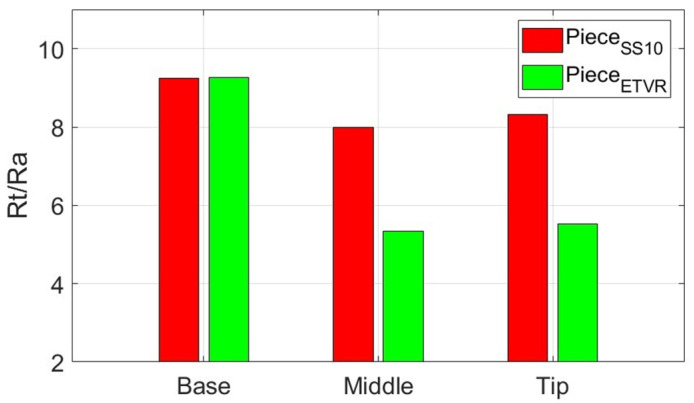
Rt/Ra ratio for surfaces formed with SS10 and ETVR tools.

When comparing the SS10 and ETVR tools, the upper limits of the variation ranges for the roughness parameters Ra and Rt of the parts formed with the SS10 tool are nearly equal to the lower limits of the same parameters for the parts formed with the ETVR tool (refer to Table 20). However, the visual quality of the surface created with the ETVR tool is superior to that of the surface made with the SS10 tool (see Figure 17 and Figure 25). The difference in performance can be attributed to the fact that the microchips produced by the ETVR tool are predominantly smaller, measuring 8 μm, compared to those made by the SS10 tool, which measure 12 μm. This size difference results in fewer scratches and local defects on the part. As a result, the Rt/Ra ratio for surfaces formed with the ETVR tool is more favorable, indicating a better alignment of the extreme values (Rt) with the average values (Ra). Consequently, the surface appears more uniform, particularly at the middle and tip of the cone (see Figure 38).

All these results and interpretations can be explained as follows:

- When using the SS5 tool, the reduced active area focuses the deforming forces on a smaller volume of material. This concentration generates higher stresses, leading to the detachment of a larger number of microchips, which ultimately damages the surface layer.
- In contrast, the SS10 tool, which has a larger contact area, distributes stresses more evenly. This even distribution reduces specific friction and prevents the accumulation of microchips. Additionally, the larger radius of the tool improves the overlap of the forming trajectory determined by the incremental step in the *Z*-axis direction.
- The asymmetric geometry of the active area of the innovative ETVR tool, along with its varying radius (between that of the SS5 tool and the SS10 tool), creates nonuniform yet controlled friction with the workpiece. This design results in intermittent radial forming, promoting the selfcleaning of the surface and helping to eliminate fine chips, a mechanism that is rarely addressed in other research.

These comparisons and explanations highlight the differences in performance and surface quality among the three tools used in the forming process. Our conclusions align with those presented in works [14,15,23,56], which demonstrate that the geometry of the tool’s active area significantly influences friction and the extent of tool abrasion against the semifinished product. Tools with a flat shape or a small radius increase sliding friction and contribute to the formation of abrasive microchips, which deteriorate surface quality.

### 4.2. Microhardness of the Surface Layer: Trends and Justifications

**Figure 39:**
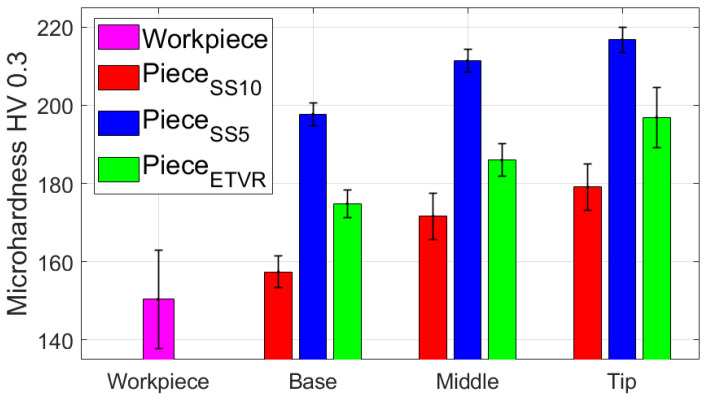
The average microhardness values HV of the parts formed with the three different tools.

The use of the three tools in the SPIF process increased the microhardness of the part when compared to the HV average microhardness of the blank, which was measured at 150.4. This increase indicates that the formed surface experienced hardening due to local plastic deformation. The results demonstrate that hardening is most pronounced at the tip of the cone, which can be attributed to a greater degree of local deformation occurring towards the end of the forming process, as illustrated in Figure 39.

The highest values of microhardness, indicating significant work hardening, were observed when using the SS5 tool. The average microhardness ranged from 197.7 HV at the base of the cone to 216.7 HV at its tip, reflecting a variation of 19 HV along the cone’s surface. In contrast, the lowest microhardness values, and consequently the least amount of work hardening, were recorded with the SS10 tool. The average microhardness for this tool ranged from 157.4 HV at the base of the cone to 179.1 HV at the tip, showing a variation of 21.7 HV along the cone’s surface.

Using the ETVR tool resulted in intermediate average microhardness values compared to those obtained with the SS10 and SS5 tools. The average microhardness values ranged from 174.8 to 196.9 HV. The variation of the average microhardness values along the generatrix was 22.1 HV, the largest among the three parts analyzed. This variation can be attributed to the tool’s variable geometry, which causes a progressive local deformation. This leads to alternating areas of high stress and local relaxation, resulting in a deeper and more uniform hardening of the surface layer.

These findings align with conclusions from studies [10,18], which demonstrate a direct relationship between local deformation and surface hardening.

The microhardness parameter HV of the parts formed using the three tools was compared using the statistical procedure outlined in Section 2 (Relations (5)–(14)). The results of this statistical analysis are presented in Table 23. The results clearly indicate that the differences in the average HV microhardness values of the parts formed with the three tools are highly statistically significant (*p* << 0.001) across all analyzed areas: Base, Middle, and Top. The following points have been noted regarding the comparisons made:

- Comparison of SS10 and SS5: The average hardness values are consistently higher for parts formed with SS5 compared to those formed with SS10. The percentage differences are 25.6% at the base, 23.14% at the middle, and 20.98% at the tip. The Cohen’s *d* value, in absolute terms, ranges from 7.35 to 10.71, which is the highest among the comparisons conducted. The SS5 tool, which has a smaller radius, results in a significantly greater hardening of the surface layer of the part. This is due to the concentration of force on a smaller surface area, which enhances the hardening effect.
- In the comparison between SS5 and ETVR, the average HV values for the parts formed with SS5 are consistently higher than those formed with ETVR. However, the percentage differences are more minor than in the previous comparison, with differences of 13.1% at the base, 13.6% in the middle, and 10.06% at the top. The Cohen’s *d* values are lower than in the earlier comparison, ranging from 3.12 to 6.73. Nevertheless, the effects remain strong in this case as well.
- The comparison between the parts formed with ETVR and SS10 tools shows significant differences (*p* << 0.001). Using the ETVR tool results in higher hardness values of the formed surface compared to the SS10 tool. The percentage differences are 11.04% at the base, 8.39% in the middle, and 9.92% at the tip. Cohen’s *d* values range from 2.42 to 4.33, indicating large to considerable effects in this case. These differences can be attributed to the geometric shape of the ETVR tool, which, due to its eccentricity and variable, intermittent contact, produces more intense work hardening compared to the 10 mm radius tool (SS10) but less than the 5 mm radius tool (SS5).

### 4.3. The Impact of Material Anisotropy on Surface Roughness and Microhardness

The material used for the workpiece, the AA6061-T6 aluminum alloy, exhibits structural anisotropy due to the rolling process. This means that the mechanical properties and surface topography vary between the rolling direction (RD) and the transverse direction (TD).

The average values of the roughness parameter Ra for the parts formed using SPIF indicate a general trend of increased roughness in the thickness direction (TD) compared to the rolling direction (RD). However, there are some local exceptions, particularly in the tip area of the parts formed with the SS5 tool. This variation can be attributed to several factors:

- The initial topography of the sheet features asperities that are oriented linearly along the RD. When deformation occurs parallel to the RD, these asperities tend to flatten and align with the tool’s feed direction, resulting in a smoother surface.
- In the TD direction, the tool intersects the laminated asperities, leading to a more disruptive mechanical interaction characterized by increased friction. This interaction can cause the formation of larger microchips and an increase in surface roughness.

The roughness parameter Rt of the SPIF-formed surfaces shows significant differences between the average values measured in two directions. Since the Rt parameter is sensitive to extreme values, such as scratches and asperities, it exhibited higher and more variable values in the radial direction (RD). This variability is attributed to the overlap of tool marks with the laminated texture. In contrast, the values recorded in the transverse direction (TD) were more uniform as the tool moved parallel to the spiral trajectory. This observation suggests that analyzing both the average roughness (Ra) and the maximum roughness (Rt) is crucial for assessing the roughness characteristics of SPIF-formed surfaces. While Ra indicates the overall surface trend, Rt highlights localized defects and anisotropic behavior.

**Figure 40:**
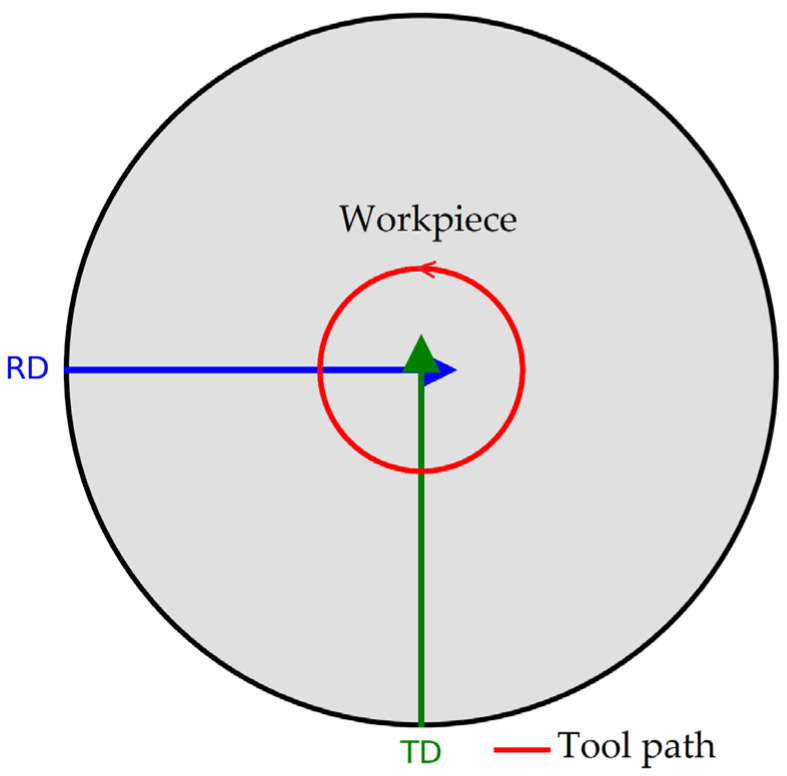
Influence of tool path on rolling and transverse directions.

In the evaluation of HV microhardness in SPIF-formed parts, the values were generally higher along the rolling direction (RD) compared to the transverse direction (TD). This suggests that grain compaction is aligned with the tool path, which is a favorable orientation for plastic deformation. Such alignment leads to local work hardening and minimizes the occurrence of intergranular microcracks. However, this trend is reversed in the cone tip area or when using the SS5 tool. This reversal can be attributed to the local concentration and redistribution of deformation stresses [18], the inclination angle of the part wall, and the tool path that alternately crosses RD and TD, as illustrated in Figure 40.

The diagram in Figure 40 illustrates how the tool path alternately intersects two key orientations of the workpiece: the rolling direction (RD) and the transverse direction (TD), from various angles. This interaction gives rise to distinct material flow behaviors during deformation, which account for the observed variations and even reversals in surface roughness and microhardness values across different regions of the workpiece.

This analysis highlights that the orientation of the workpiece in relation to the part geometry—particularly the alignment of the rolling direction—is not a minor detail. Instead, it is a crucial experimental parameter that must be carefully studied, controlled, and integrated into SPIF optimization strategies to ensure consistent, high-quality results.

### 4.4. Integrated Analysis: Balancing Surface Roughness and Microhardness

**Figure 41:**
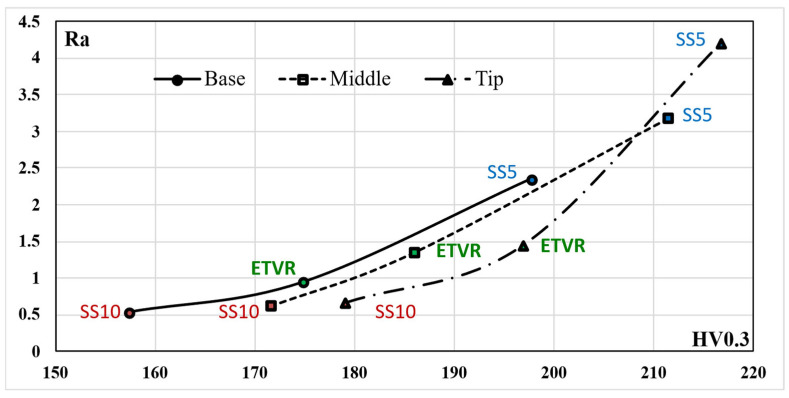
Correlation between the roughness parameter Ra values and the microhardness HV measurements taken in three specific areas along the cone’s generatrix for parts formed using the three different tools.

**Figure 42:**
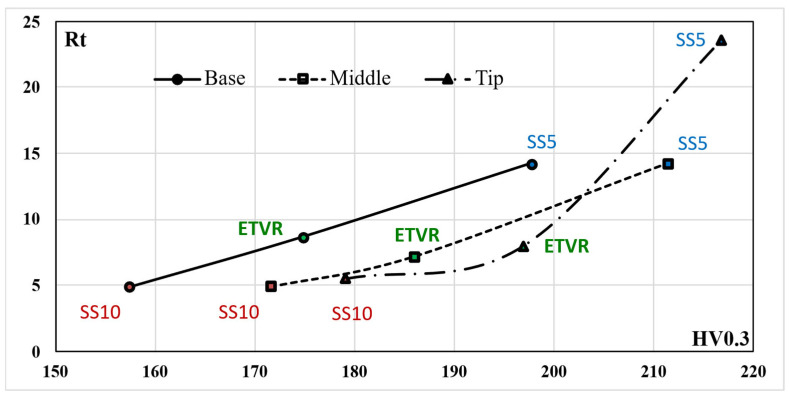
Correlation between the roughness parameter Rt values and the microhardness HV measurements taken in three specific areas along the cone’s generatrix for parts formed using the three different tools.

The representation of the average roughness values, Ra and Rt, for the parts formed using the three tools is presented in Figure 41 and Figure 42. These values are related to the microhardness measurements taken from the same areas of the cone’s generatrix.

The following observations arise from the analysis of the two figures:

- The graph in Figure 41 shows a clear trend: as the microhardness (HV) increases, the average roughness (Ra) also tends to grow in a roughly linear fashion. This indicates that the more pronounced asperities on the formed surface (higher Ra) are associated with greater deformation of the material in the surface layer of the part. This phenomenon is linked to grain deformation and an increase in microhardness.
- The graph in Figure 42 indicates that the roughness parameter Rt tends to increase with the rise in microhardness HV. However, this relationship is not linear for parts formed using the SS5 tool. This can be attributed to the appearance of deep scratches during the forming process with the SS5 tool, which do not necessarily result in hardening. As a result, the increase in HV is not proportional to the increase in Rt.
- The Rt parameter focuses on the extremes of the surface profile and is sensitive to isolated defects, which do not accurately reflect the uniformity of the deformation process. Therefore, it is considered that the correlation between Ra and HV is more reliable than that between Rt and HV.

From the analysis of these correlations, some relevant conclusions for the research undertaken result:

- SPIF forming with the SS10 tool leads to obtaining smooth and uniform surfaces, but produces moderate hardening of the material.
- SPIF forming with the SS5 tool leads to obtaining rough surfaces, with possible extreme defects, but with intense hardening of the material.
- SPIF forming with the ETVR tool leads to obtaining surfaces that present a compromise between surface quality and material hardening.

## 5. Conclusions

This research investigated the impact of tool geometry on the surface quality of AA6061-T6 aluminum alloy parts produced by Single Point Incremental Forming. A comparative evaluation was conducted using two conventional hemispherical tools with radii of 5 mm and 10 mm, as well as an innovative eccentric tool with a variable radius (ETVR). The analysis focused on several factors, including surface roughness (Ra and Rt), microhardness (HV), microchip formation, and the visual appearance of the surfaces. Measurements were taken from three key regions of the cone: the base, middle, and tip, in both the rolling (RD) and transverse (TD) directions. Statistical analysis was applied to validate the observed differences. The study also emphasized the influence of material anisotropy on the measured values. By combining experimental measurements with statistical and causal interpretations, the research aimed to clarify the trade-offs between surface finish and work hardening.

The findings of this study reinforce existing literature’s conclusions regarding the relationship between tool geometry, process mechanics, and surface integrity. Additionally, the findings reveal significant performance differences among the tools, consistent with existing literature.

This research provides original insights by evaluating the novel Eccentric Tool with Variable Radius (ETVR), which outperformed traditional hemispherical tools regarding surface integrity and mechanical performance.

The specific findings of the study are summarized as follows:

1. The tool with the smallerradius hemispherical head (SS5) resulted in the highest surface roughness values. This is due to increased contact and the accumulation of microchips in the deformation zone. However, it also caused the most significant work hardening, which is a result of more intense localized plastic deformation.
2. The tool with the largerradius hemispherical head (SS10) produced the best surface finish due to its increased contact area and overlapping tool paths, but it also led to the least amount of work hardening.
3. The innovative ETVR tool demonstrated impressive performance by producing a low surface roughness, attaining high and uniform microhardness, and effectively accommodating complex deformations, especially in the cone tip region. As a result, this tool serves as a viable alternative to conventional hemispherical tools, providing clear advantages for industrial applications.
4. Material anisotropy, which refers to the differences between the rolling direction (RD) and transverse direction (TD), significantly affected both surface roughness and microhardness. In some cases, reversals between these directions were observed, which can be explained by the part geometry, tool path direction, and local stress accumulations.

The limitations of this study arise from two main factors. First, the performance of the three tools was assessed using only one type of material. Second, a single set of technological parameters was applied in the evaluation.

The study can be expanded in the future by carrying out the following:

- Evaluating the performance of the innovative tool on different materials with varying ductility, such as aluminum alloys AA7075 and AA1050;
- Analyzing the tool’s performance under different technological parameters (such as tool speed, feed rate, and incremental step) to differentiate the effects of tool geometry from any potential synergies with these parameters.

## Figures and Tables

**Table 1:** Chemical composition of the AA6061 alloy.

Chemical Element	Si	Cu	Mg	Cr	Fe	Mn	Zn	Ti	Other	Al
Effective values (wt%)	1.52	0.38	1.59	0.27	0.65	0.19	0.1	0.04	1.05	94.21
Measurement error (%)	0.010	0.001	0.012	0.003	0.003	0.002	0.001	0.005	-	0.08

**Table 2:** Mechanical properties of the AA6061 alloy.

Mechanical Property	Value According to UNS A96061 [40]	Effective Value, in a Direction at 45° to RD
Tensile strength, Rm [MPa]	310	329.77
Yield strength, Rp0.2 [MPa]	276	282.65
Elongation, At [%]	12–17	13.85
Poisson’s ratio, ν [-]	0.33	0.33
Modulus of elasticity, E [GPa]	68.9	64.83

**Table 3:** Parameters used in roughness measurement.

Piece	Ra (μm)	λc (mm)	N (Cut-Off nb.)	Lt (mm)	v (mm/s)	F (mN)
Blank	0.1–0.2	0.8	4	3.2	0.25	0.75
SS10						
ETVR						
SS5	2.0–10.0	2.5	1	2.5		

**Table 4:** Experimental research plan to measure the quality parameters Ra, Rt, and HV of surfaces formed by SPIF.

Tool	Technological Parameter			Rolling Direction		Transverse Direction	
	n (rot/min)	v (mm/min)	Δz (mm)	E2	E4	E1	E3
SS10	2000	1000	0.3	Areas: Base, Middle, Tip 5 measurements in each area			
SS5							
ETVR							

**Table 5:** Statistical values of the roughness parameter Ra for the blank.

Direction	Average Ra (μm)	Standard Deviation	RSD (%)	Margin of Error
RD	0.115	0.005	4.416	0.006
TD	0.454	0.012	2.776	0.016
Blank average	0.284	0.178	59.632	0.128

**Table 6:** Statistical values of the roughness parameter Rt for the blank.

Direction	Average Rt (μm)	Standard Deviation	RSD (%)	Margin of Error
RD	1.381	0.349	25.264	0.430
TD	2.488	0.116	4.649	0.140
Blank average	1.900	0.633	33.297	0.450

**Table 7:** Average Ra roughness values measured at regions B, M, and T from samples formed using the SS10 tool, along with relevant statistical data.

Direction	Area	Average Ra (μm)	Standard Deviation	RSD (%)	Margin of Error
RD average (E2 and E4)	Base	0.500	0.020	4.050	0.014
	Middle	0.599	0.026	4.464	0.019
	Tip	0.641	0.025	3.929	0.018
TD average (E1 and E3)	Base	0.564	0.026	4.768	0.019
	Middle	0.626	0.040	6.383	0.029
	Tip	0.678	0.035	5.178	0.025
Piece average (E1–E4)	Base	0.532	0.040	7.514	0.019
	Middle	0.613	0.036	5.868	0.017
	Tip	0.660	0.035	5.370	0.017

**Table 8:** Average Rt roughness values measured at regions B, M, and T from samples formed using the SS10 tool, along with relevant statistical data.

Sample	Area	Average Rt (μm)	Standard Deviation	RSD (%)	Margin of Error
RD average (E2 and E4)	Base	5.049	1.036	20.527	0.741
	Middle	4.161	0.563	13.542	0.403
	Tip	5.471	0.919	16.795	0.657
TD average (E1 and E3)	Base	4.793	1.214	25.324	0.868
	Middle	5.641	0.998	17.685	0.714
	Tip	5.506	0.784	14.235	0.561
Piece average (E1–E4)	Base	4.921	1.106	22.482	0.518
	Middle	4.901	1.095	22.335	0.512
	Tip	5.489	0.831	15.147	0.389

**Table 9:** Rt/Ra ratio values for points of interest (B, M, and V) and the directions specific to the area formed with the SS10 tool.

Area	RD (Average)	TD (Average)	Piece (Average)
Base	10.098	8.498	9.250
Middle	6.947	9.011	7.995
Tip	8.535	8.121	8.317

**Table 10:** Average Ra roughness values measured at regions B, M, and T from samples formed using the SS5 tool, along with relevant statistical data.

Sample	Area	Average Ra (μm)	Standard Deviation	RSD (%)	Margin of Error
RD average (E2 and E4)	Base	2.335	0.109	4.680	0.078
	Middle	3.299	0.091	2.772	0.065
	Tip	4.339	0.137	3.165	0.098
TD average (E1 and E3)	Base	2.357	0.066	2.826	0.048
	Middle	3.044	0.104	3.424	0.075
	Tip	4.049	0.130	3.209	0.093
Piece average (E1–E4)	Base	2.346	0.088	3.784	0.042
	Middle	3.172	0.161	5.102	0.076
	Tip	4.194	0.197	4.716	0.093

**Table 11:** Average Rt roughness values measured at regions B, M, and T from samples formed using the SS5 tool, along with relevant statistical data.

Sample	Area	Average Rt (μm)	Standard Deviation	RSD (%)	Margin of Error
RD average (E2 and E4)	Base	14.332	3.048	21.265	2.180
	Middle	14.808	1.894	12.790	1.355
	Tip	25.852	4.293	16.605	3.071
TD average (E1 and E3)	Base	14.093	1.963	13.930	1.404
	Middle	13.669	1.491	10.911	1.067
	Tip	21.205	4.502	21.231	3.221
Piece average (E1–E4)	Base	14.213	2.498	17.576	1.169
	Middle	14.239	1.759	12.354	0.823
	Tip	23.529	4.900	20.826	2.293

**Table 12:** Rt/Ra ratio values for points of interest (B, M, and V) and the directions specific to the area formed with the SS5 tool.

Area	RD (Average)	TD (Average)	Piece (Average)
Base	6.138	5.979	6.058
Middle	4.489	4.490	4.489
Tip	5.958	5.237	5.610

**Table 13:** Average Ra roughness values measured at regions B, M, and T from samples formed using the ETVR tool, along with relevant statistical data.

Sample	Area	Average Ra (μm)	Standard Deviation	RSD (%)	Margin of Error
RD average (E2 and E4)	Base	0.952	0.035	3.706	0.025
	Middle	1.336	0.050	3.748	0.036
	Tip	1.446	0.049	3.433	0.036
TD average (E1 and E3)	Base	0.926	0.036	3.891	0.026
	Middle	1.358	0.031	2.319	0.023
	Tip	1.441	0.050	3.503	0.036
Piece average (E1–E4)	Base	0.939	0.0371	3.960	0.017
	Middle	1.347	0.042	3.146	0.020
	Tip	1.444	0.048	3.381	0.023

**Table 14:** Average Rt roughness values measured at regions B, M, and T from samples formed using the ETVR tool, along with relevant statistical data.

Sample	Area	Average Rt (μm)	Standard Deviation	RSD (%)	Margin of Error
RD average (E2 and E4)	Base	9.455	1.992	21.073	1.425
	Middle	7.003	1.929	27.548	1.380
	Tip	8.052	2.543	31.583	1.819
TD average (E1 and E3)	Base	7.932	1.118	14.092	0.800
	Middle	7.389	2.195	29.707	1.570
	Tip	7.888	1.473	18.678	1.054
Piece average (E1–E4)	Base	8.694	1.756	20.195	0.822
	Middle	7.196	2.021	28.086	0.946
	Tip	7.970	2.025	25.402	0.947

**Table 15:** Rt/Ra ratio values for points of interest (B, M, and V) and the directions specific to the area formed with the ETVR tool.

Area	RD (Average)	TD (Average)	Piece (Average)
Base	9.932	8.566	9.259
Middle	5.242	5.441	5.342
Tip	5.568	5.474	5.519

**Table 16:** Statistical values of the microhardness HV of the blank.

Direction	Average HV 0.3	Standard Deviation	RSD (%)	Margin of Error
RD	158.58	1.427	0.900	1.77
TD	142.24	1.906	1.340	2.37
Blank average	150.4	8.757	5.822	6.26

**Table 17:** Average HV microhardness values measured at regions B, M, and T from samples formed using the SS10 tool, along with relevant statistical data.

Sample	Area	Average HV 0.3	Standard Deviation	RSD (%)	Margin of Error
RD average (E2 and E4)	Base	156.8	5.545	3.536	3.97
	Middle	175.9	5.087	2.892	3.64
	Tip	180.9	8.280	4.577	5.92
TD average (E1 and E3)	Base	158	2.794	1.768	2.00
	Middle	167.3	4.244	2.537	3.04
	Tip	177.3	2.897	1.634	2.07
Piece average (E1–E4)	Base	157.4	4.316	2.742	2.02
	Middle	171.6	6.323	3.685	2.96
	Tip	179.1	6.321	3.529	2.96

**Table 18:** Average HV microhardness values measured at regions B, M, and T from samples formed using the SS5 tool, along with relevant statistical data.

Sample	Area	Average HV 0.3	Standard Deviation	RSD (%)	Margin of Error
RD average (E2 and E4)	Base	198.5	2.506	1.262	1.79
	Middle	210.2	2.919	1.388	2.09
	Tip	215.1	2.894	1.345	2.07
TD average (E1 and E3)	Base	196.9	3.581	1.819	2.56
	Middle	212.5	2.784	1.310	1.99
	Tip	218.3	3.394	1.554	2.43
Piece average (E1–E4)	Base	197.7	3.113	1.574	1.46
	Middle	211.4	3.027	1.431	1.42
	Tip	216.7	3.498	1.614	1.64

**Table 19:** Average HV microhardness values measured at regions B, M, and T from samples formed using the ETVR tool, along with relevant statistical data.

Sample	Area	Average HV 0.3	Standard Deviation	RSD (%)	Margin of Error
RD average (E2 and E4)	Base	173.1	2.656	1.534	1.90
	Middle	183.1	2.692	1.470	1.93
	Tip	189.3	2.846	1.503	2.04
TD average (E1 and E3)	Base	176.4	3.927	2.226	2.81
	Middle	188.9	3.998	2.116	2.86
	Tip	204.5	2.838	1.388	2.03
Piece average (E1–E4)	Base	174.8	3.674	2.101	1.72
	Middle	186	4.466	2.401	2.09
	Tip	196.9	8.254	4.192	3.86

**Table 20:** The relationship between the size of microchips and the surface roughness parameters of parts formed by SPIF.

Tool	Microchip Size [μm]		Roughness Limits [μm]	
	Limits	Average	Ra	Rt
SS10	7–18	12	0.500–0.678	4.161–5.641
SS5	21–83	45	2.335–4.339	13.669–25.852
ETVR	2–51	8	0.926–1.446	7.003–9.455

**Table 21:** Results of the statistical analysis using the two-sample *t*-test for the roughness parameter Ra.

Tested Sample	Area	*n*	*X_m_*	*s*	*s* ^2^	s12s22	*p*-Value	Cohen’s *d*	Δ%
SS10–SS5	Base	20	0.532	0.040	0.002	0.203	1.68 × 10^−33^	−26.35	341.17
		20	2.346	0.089	0.008				
	Middle	20	0.613	0.036	0.001	0.049	4.02 × 10^−26^	−21.83	417.65
		20	3.171	0.162	0.026				
	Tip	20	0.659	0.035	0.001	0.032	1.26 × 10^−26^	−24.88	536.06
		20	4.194	0.198	0.039				
SS5–ETVR	Base	20	2.346	0.089	0.008	5.701	6.89 × 10^−30^	20.66	149.72
		20	0.939	0.037	0.001				
	Middle	20	3.171	0.162	0.026	14.581	1.39 × 10^−23^	15.42	135.45
		20	1.347	0.042	0.002				
	Tip	20	4.194	0.198	0.039	16.415	2.6 × 10^−25^	19.09	190.58
		20	1.443	0.049	0.002				
ETVR–SS10	Base	20	0.939	0.037	0.001	0.865	9.06 × 10^−30^	10.56	76.66
		20	0.532	0.040	0.002				
	Middle	20	1.347	0.042	0.002	1.388	5.32 × 10^−39^	18.68	119.86
		20	0.613	0.036	0.001				
	Tip	20	1.443	0.049	0.002	1.897	9.84 × 10^−39^	18.38	118.90
		20	0.659	0.035	0.001				

**Table 22:** Results of the statistical analysis using the two-sample *t*-test for the roughness parameter Rt.

Tested Sample	Area	*n*	*X_m_*	*s*	*s* ^2^	s12s22	*p*-Value	Cohen’s *d*	Δ%
SS10–SS5	Base	20	4.921	1.106	1.224	0.196	1.64 × 10^−14^	−4.81	188.82
		20	14.213	2.498	6.240				
	Middle	20	4.901	1.095	1.198	0.387	6.89 × 10^−22^	−6.37	190.54
		20	14.239	1.759	3.094				
	Tip	20	5.488	0.831	0.691	0.029	5.12 × 10^−13^	−5.13	328.70
		20	23.529	4.900	24.012				
SS5–ETVR	Base	20	14.213	2.498	6.240	2.024	8.83 × 10^−10^	2.56	63.49
		20	8.693	1.756	3.083				
	Middle	20	14.239	1.759	3.094	0.758	3.17 × 10^−14^	3.72	97.88
		20	7.196	2.021	4.085				
	Tip	20	23.529	4.900	24.012	5.859	8.56 × 10^−13^	4.15	195.22
		20	7.970	2.025	4.099				
ETVR–SS10	Base	20	8.693	1.756	3.083	2.519	7.73 × 10^−10^	2.57	76.65
		20	4.921	1.106	1.224				
	Middle	20	7.196	2.021	4.085	3.409	1.10 × 10^−5^	1.41	46.83
		20	4.901	1.095	1.198				
	Tip	20	7.970	2.025	4.099	5.929	3.03 × 10^−5^	1.60	45.21
		20	5.488	0.831	0.691				

**Table 23:** Results of the statistical analysis using the two-sample *t*-test for the microhardness parameter HV.

Tested Sample	Area	*n*	*X_m_*	*s*	*s* ^2^	s12s22	*p*-Value	Cohen’s *d*	Δ%
SS10–SS5	Base	20	157.390	4.317	18.633	1.922	5.45 × 10^−30^	−10.71	25.60
		20	197.680	3.113	9.693				
	Middle	20	171.610	6.323	39.986	4.364	1.72 × 10^−20^	−8.01	23.14
		20	211.325	3.027	9.163				
	Tip	20	179.115	6.321	39.959	3.265	1.37 × 10^−20^	−7.35	20.98
		20	216.685	3.498	12.239				
SS5–ETVR	Base	20	197.680	3.113	9.693	0.718	1.03 × 10^−22^	6.73	13.11
		20	174.765	3.674	13.499				
	Middle	20	211.325	3.027	9.163	0.459	1.68 × 10^−22^	6.64	13.61
		20	186.005	4.467	19.952				
	Tip	20	216.685	3.498	12.239	0.180	3.18 × 10^−10^	3.12	10.06
		20	196.880	8.254	68.136				
ETVR–SS10	Base	20	174.765	3.674	13.499	0.724	2.74 × 10^−16^	4.33	11.04
		20	157.390	4.317	18.633				
	Middle	20	186.005	4.467	19.952	0.499	4.42 × 10^−10^	2.63	8.39
		20	171.610	6.323	39.986				
	Tip	20	196.880	8.254	68.136	1.705	3.39 × 10^−9^	2.42	9.92
		20	179.115	6.321	39.959				

## Data Availability

The original contributions presented in the study are included in the article, further inquiries can be directed to the corresponding author.
